# Model fit versus biological relevance: Evaluating photosynthesis-temperature models for three tropical seagrass species

**DOI:** 10.1038/srep39930

**Published:** 2017-01-04

**Authors:** Matthew P. Adams, Catherine J. Collier, Sven Uthicke, Yan X. Ow, Lucas Langlois, Katherine R. O’Brien

**Affiliations:** 1 School of Chemical Engineering, The University of Queensland, St Lucia, 4072, Australia; 2 College of Marine and Environmental Science, James Cook University, Townsville, 4811, Australia; 3 Centre for Tropical Water & Aquatic Ecosystem Research (TropWATER), James Cook University, Cairns, 4870, Australia; 4 Australian Institute of Marine Science, PMB No. 3, Townsville, 4811, Australia; 5 Experimental Marine Ecology Laboratory, Department of Biological Sciences, National University of Singapore, 117557, Singapore

## Abstract

When several models can describe a biological process, the equation that best fits the data is typically considered the best. However, models are most useful when they also possess biologically-meaningful parameters. In particular, model parameters should be stable, physically interpretable, and transferable to other contexts, e.g. for direct indication of system state, or usage in other model types. As an example of implementing these recommended requirements for model parameters, we evaluated twelve published empirical models for temperature-dependent tropical seagrass photosynthesis, based on two criteria: (1) goodness of fit, and (2) how easily biologically-meaningful parameters can be obtained. All models were formulated in terms of parameters characterising the thermal optimum (*T*_*opt*_) for maximum photosynthetic rate (*P*_max_). These parameters indicate the upper thermal limits of seagrass photosynthetic capacity, and hence can be used to assess the vulnerability of seagrass to temperature change. Our study exemplifies an approach to model selection which optimises the usefulness of empirical models for both modellers and ecologists alike.

For relatively simple biological process rates, several empirical models may adequately describe the process rate’s dependence on environmental factors^1^. The best model is then typically chosen based on goodness of fit^2^.

However, the selected model may not be particularly informative if its parameters have no intrinsic biological meaning. Platt *et al*.^3^ and Jassby & Platt^4^ suggested that parameters of models fitted to biological processes should be both stable (well constrained and mutually independent) and physically interpretable. For greatest utility, we suggest that the model parameters should also be *transferable*. Parameters that are transferable have usage beyond the selected empirical model; they may be directly compared to experimentally measurable quantities to provide an indication of system state, and/or easily used in different model types. For example, the minimum light requirement (MLR) of seagrass is a transferable parameter, as comparison of local light levels to MLR indicates whether seagrass are at risk of loss due to light deprivation^5^,^6^, and MLR can be used to parameterise both mechanistic^7^ and statistical^8^,^9^ coastal ecosystem models. Model parameters that are (1) stable, (2) physically interpretable and (3) transferable have the greatest biological meaning, and therefore we define parameters that satisfy these three criteria as *biologically-meaningful*.

In this paper, we demonstrate a model selection procedure that gives similar importance to goodness of fit and obtaining biologically-meaningful parameters. To this end, aquatic plant photosynthesis is a biological process which has a well-established dependence on temperature, but the parameterisation of this process is not yet standardised. Aquatic plant photosynthetic rates rise gradually with temperature up to a thermal optimum^10^,^11^ and sharply decrease at higher temperatures^12^. This photosynthesis-temperature relationship has been previously fitted to exponential^13^, Gaussian^11^,^14^, and enzyme kinetics-based functions^15^. Of these functions, only the enzyme kinetics-based function captures the shape of the photosynthesis-temperature curve over the full range of temperatures^16^, including the sharp decline in photosynthetic rates expected at high temperatures. However, the enzyme kinetics-based function is not written in terms of biologically-meaningful parameters^17^, so may not be the best model for capturing the dependence of photosynthesis on temperature.

The purpose of this study was to apply a model selection approach that gives similar importance to fitting the data and obtaining biological-meaningful parameters, to identify the best model for the dependence of aquatic plant photosynthesis on temperature. Twelve models (Table 1) were fitted to seven photosynthesis-temperature curves for seagrass. The photosynthesis-temperature curves were obtained from three tropical seagrass species (*Cymodocea serrulata, Halodule uninervis* and *Zostera muelleri*) growing at two different locations on the eastern coast of Queensland, Australia (Green Island and Moreton Bay) in two different seasons (summer and winter). The models were formulated in terms of as many biologically-meaningful parameters as possible, including the thermal optima (*T*_*opt*_) and maxima (*T*_max_) for seagrass photosynthesis. These two critical temperatures can be compared to seawater temperatures in areas where tropical seagrass currently colonise, to provide an indication of the vulnerability of these seagrasses to ocean warming^18^. Specifically, thermal optima for seagrass photosynthesis are expected to be higher than thermal optima for growth^19^, due to the synergistic effects of increased respiration and sulphide intrusion at elevated temperatures^20^,^21^; thus, seagrass present in water temperatures close to *T*_*opt*_ and *T*_max_ for photosynthesis are likely to be at risk of heat stress-induced decline. The model selection approach employed here can also be applied in several other biological contexts to increase the utility of fitted models, and encourage standardisation of model parameterisations for well-understood biological processes^1^.

## Results

**Figure 1:**
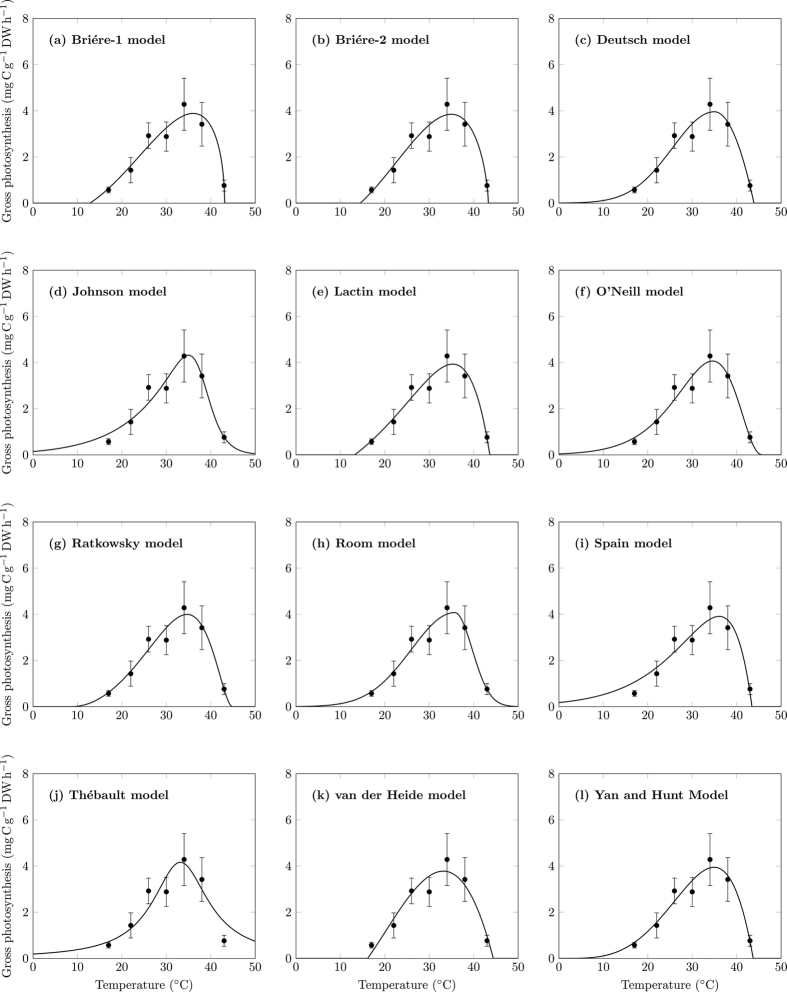
All 12 models fitted to the photosynthesis-temperature curve of *C. serrulata* at Green Island in summer. Error bars indicate ±SD.

All 12 models were successfully fitted to all seven photosynthesis-temperature curves of seagrass using nonlinear regression. The fit of all 12 models to the photosynthesis-temperature curve of *C. serrulata* in summer at Green Island is shown in Fig. 1, and the fit of the models to the other six photosynthesis-temperature curves is shown in Supplementary Figures S2–S7. Parameters identified for the fit of all models to all photosynthesis-temperature curves are listed in Supplementary Table S1.

**Figure 2:**
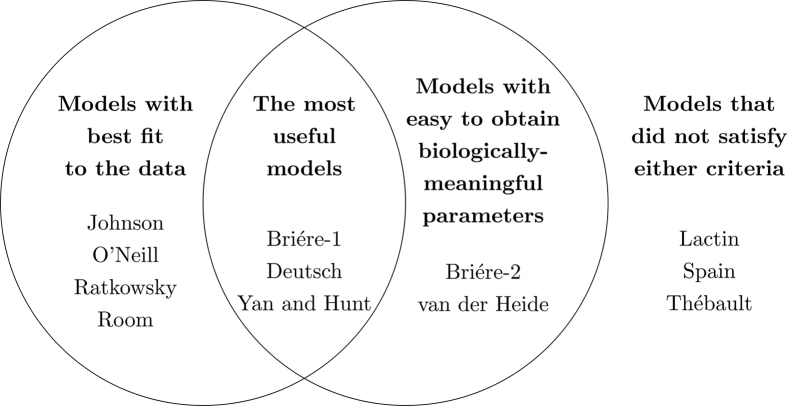
Criteria for selecting the best model, and how the 12 tested models satisfied these criteria.

We next evaluated all 12 models to identify which model was the best. To identify the best empirical model for fitting the temperature-dependence of aquatic plant photosynthesis, we used two criteria: (1) goodness of fit, by comparing four statistical metrics (Akaike weight, Schwarz weight, R^2^ and refined index of agreement), and (2) the ease at which biologically-meaningful parameters, specifically *T*_*opt*_, *T*_max_ and *P*_max_, can be obtained (Fig. 2).

### Best models, based on goodness of fit

Akaike and Schwarz weights were better than the other two statistical metrics (adjusted R^2^ and refined index of agreement) at identifying differences between model fits to the data. We came to this conclusion because adjusted R^2^ and refined index of agreement values varied more substantially between treatments than between models, whilst Akaike and Schwarz weights varied more substantially between models than between treatments. Specifically, when averaged across the seven treatments, all 12 models produced similar mean adjusted R^2^ values and refined indices of agreement (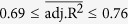; 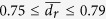). However, there was far more variability in adjusted R^2^ values and refined indices of agreement for each of the seven treatments when averaged across the 12 models (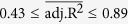; 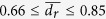). In contrast, when averaged over the seven treatments, Akaike and Schwarz weights varied substantially between the 12 models (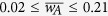; 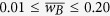). There was no difference in Akaike and Schwarz weights between treatments when averaged across all models because these metrics are indicators of relative performance and therefore trivially give exactly 1/12 so that they add to unity over the 12 models.

We therefore identified the best fitting models based on mean Akaike and Schwarz weights (averaged over the seven treatments), and for each of these two metrics we identified the six best models. The six best models based on mean Akaike weight, ranked from first to sixth, were the Johnson, Room, O’Neill, Yan and Hunt, Ratkowsky, and Deutsch models (Table 2). The six best models based on mean Schwarz weight, ranked from first to sixth, were the Johnson, Yan and Hunt, Briére-1, Room, O’Neill, and Ratkowsky models (Table 3). On first glance, this suggests that the Johnson model may be the best model based on goodness of fit. However, this conclusion is biased by the dataset for *C. serrulata* in winter at Moreton Bay, for which the Johnson model performed especially well and obtained *w*_*A*_ and *w*_*B*_ values greater than 0.8. In contrast, *w*_*A*_ and *w*_*B*_ values were less than 0.5 for all other datasets and models.

Temporarily excluding the dataset for *C. serrulata* in winter at Moreton Bay, and recalculating the mean *w*_*A*_ and *w*_*B*_ values for each model averaged over the remaining six treatments, yields the same six best models for mean Akaike and Schwarz weights, but in a different order (Tables 2 and 3). Hence, regardless of whether the dataset for *C. serrulata* in winter at Moreton Bay is excluded or not, the best six models based on either mean Akaike weight or mean Schwarz weight were unchanged. We kept for consideration as the best model, based on goodness fit, only those models which were either one of the best six models based on mean Akaike weight or one of the best six models based on mean Schwarz weight. This yields that the seven best models based on goodness of fit were, in no particular order: the Briére-1, Deutsch, Johnson, O’Neill, Ratkowsky, Room, and Yan and Hunt models.

### Best models, based on the ease of obtaining biologically-meaningful parameters

Five of the 12 models could be easily used to obtain biologically-meaningful parameters, as follows. Three models (Lactin, Ratkowsky and Spain) were ruled out because they required solution of analytically intractable equations to obtain biologically-meaningful parameters. Three other models (Johnson, Room and Thébault) cannot predict the maximum temperature (Table 1), which is an important parameter to identify due to the potential impact of climate change on seagrass distribution^18^. The O’Neill model also could not satisfactorily estimate the maximum temperature for two of the seven treatments (Supplementary Table S1). The remaining five models (Briére-1, Briére-2, Deutsch, van der Heide, and Yan and Hunt) could predict the optimum temperature, maximum photosynthesis rate, and maximum temperature, and have simple analytical forms. Less than half of the 12 models can predict the minimum temperature (Table 1); however, globally, aquatic plants are more greatly threatened by elevated temperatures than cold temperatures^22^,^23^,^24^,^25^, so we considered the minimum temperature to be a less important parameter to estimate than the optimum and maximum temperatures. Hence, we kept for consideration the Briére-1, Briére-2, Deutsch, van der Heide, and Yan and Hunt models as the best models based on the ease of obtaining biologically-meaningful parameters.

### The Yan and Hunt model is the best model, based on both criteria

We next identified which of the 12 models satisfied both criteria, from (1) the seven best models based on goodness of fit and (2) the five best models based on obtaining biologically-meaningful parameters (Fig. 2). Three models satisfied both criteria - the Briére-1, Deutsch, and Yan and Hunt models - so these three models were further compared, based on goodness of fit and their parameters, to identify the best model. The Briére-1, and Yan and Hunt models are written *only* in terms of biologically-meaningful parameters *P*_max_, *T*_*opt*_ and *T*_max_, whilst the Deutsch model requires one additional shape parameter *a* (Supplementary Section S1). Based on goodness of fit, the Yan and Hunt model is always better than both the Briére-1 and Deutsch models, for both Akaike and Schwarz weights, whether or not the dataset for *C. serrulata* in winter at Moreton Bay is excluded or not (Tables 2 and 3).

**Figure 3:**
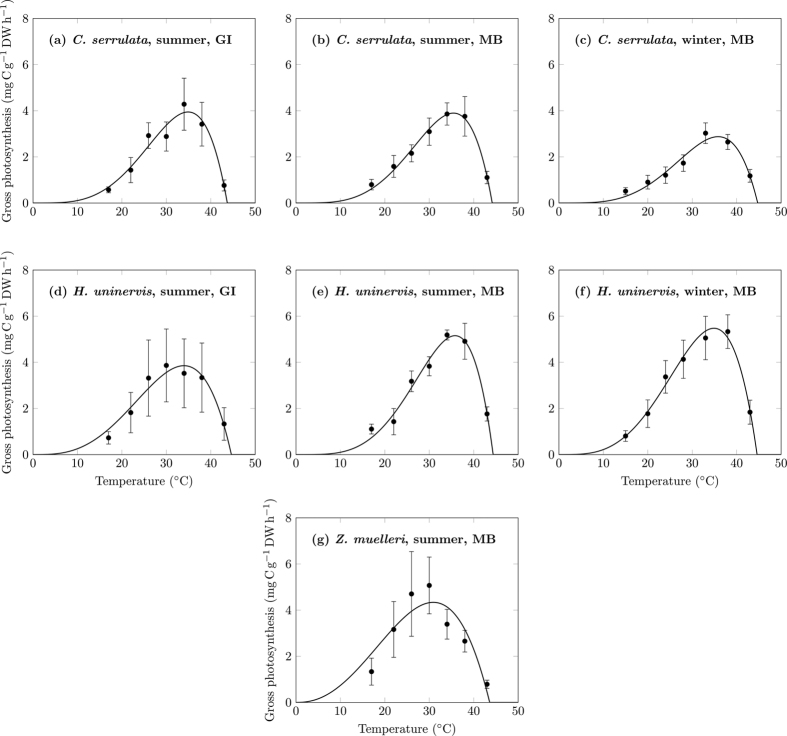
Fitting the Yan and Hunt model to the seven photosynthesis-temperature curves of tropical seagrass. GI = Green Island, MB = Moreton Bay. Error bars indicate ±SD.

Thus the Yan and Hunt model is the best of the 12 models considered here, when accounting for both the ease at which biologically-meaningful parameters can be obtained, and achieving goodness of fit between model and data. The fit of the Yan and Hunt model to all seven photosynthesis-temperature curves is shown in Fig. 3, and the parameters obtained from this model fitting are listed in Table 4.

## Discussion

Based on goodness of fit to the seven treatments, and the ease at which biologically-meaningful parameters could be obtained (Fig. 2), the best model for seagrass photosynthesis was the Yan and Hunt model^26^,


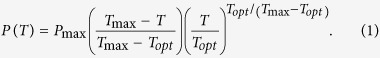


In this equation *P(T*) is the photosynthesis rate at temperature *T, P*_max_ is the maximum photosynthesis rate which occurs at the optimum temperature *T*_*opt*_, and *T*_max_ is the maximum temperature, at which the photosynthesis rate is zero. Fitting the Yan and Hunt model to the data provides parameters *P*_max_, *T*_*opt*_ and *T*_max_, which can then be used independently of the model. The shape parameter *Q*_10_, which represents the factor increase in photosynthesis rate due to a temperature increase of 10 °C at temperatures below *T*_*opt*_, can then be found by fitting equation (2) to the temperature data that is less than the optimum, *T* < *T*_*opt*_, where *T*_*opt*_ was found by fitting the Yan and Hunt model to the data.

The four parameters *P*_max_, *T*_*opt*_, *T*_max_ and *Q*_10_ are all stable, physically interpretable, and transferable, which are the three requirements for our definition of biologically-meaningful parameters. First, in terms of stability, uncertainty bounds calculated for these parameters indicated that they were well-constrained (see Table 4), and there was no obvious mutual dependence of the Yan and Hunt model parameters. Second, all four parameters have clear physical interpretations, based on their definitions provided previously in this section: *P*_max_ is the maximum photosynthesis rate which occurs at the temperature *T*_*opt*_, *T*_max_ is the maximum temperature at and above which the photosynthesis rate becomes negligible, and, for temperatures below *T*_*opt*_, *Q*_10_ is the factor increase in photosynthesis rate due to a temperature increase of 10 °C.

We have higher confidence in our estimates for thermal optima (*T*_*opt*_) than our estimates for thermal maxima (*T*_max_), because thermal optima were obtained by interpolation of the data, whilst thermal maxima were obtained from extrapolation of the data and were relatively sensitive to the gross photosynthesis rate measured at one temperature (43 °C) above the optimum. For future investigations of seagrass photosynthesis-temperature curves, more accurate estimates of *T*_max_ can be obtained by measuring gross photosynthesis rates at several temperatures above *T*_*opt*_.

Finally, the four parameters are all transferable, as they can be applied in other contexts. *T*_*opt*_ and *T*_max_ characterise temperatures above which seagrass is vulnerable to heat stress^12^; close to these temperatures, small changes in water temperature can substantially alter the ecological function of seagrass meadows^27^ over timescales potentially as small as a few days^28^. *P*_max_ estimates maximum productivity, which can be used to compare seagrass growth traits between different species^29^. Our obtained *Q*_10_ values for photosynthesis experimentally verify the hypothesis that *Q*_10_ values generally range between 2 and 3^30^, an assumption which is applied in coastal ecological models^31^. Several empirical photosynthesis-temperature models include *P*_max_, *T*_*opt*_ and *T*_max_ as parameters (Table 1), so these parameters are transferable to empirical photosynthesis-temperature models other than the Yan and Hunt model, and could also be implemented in plant growth models that are based on carbon balance^32^. Ecological implications of our results will be discussed further in a subsequent publication.

### Limitations of the Yan and Hunt model

One limitation of the Yan and Hunt model is that the minimum temperature is assumed to be zero. We do not consider this to be a major limitation, because (1) data may not be available at low temperatures, and (2) local and global ocean warming is a greater threat to seagrass distribution than cold temperatures^22^,^23^,^24^,^25^, so identifying the temperature-dependence of biological rates near the optimum and maximum temperatures is of primary importance. To consider cases where accurate modelling of low temperatures is required^33^, a four-parameter Yan and Hunt model can instead be used, which includes *T*_min_ as a parameter and is defined in equation (4) of Yan *et al*.^26^. However, we recommend that the four-parameter Yan and Hunt model should only be used if the data available for model fitting covers a wider temperature range with a larger number of different temperature values than measured here (15 to 43 °C, 7 temperature values), because data over a wider temperature range would be required for correct model fit.

A second limitation of the Yan and Hunt model is that it is not well suited to modelling positively skewed distributions: this issue was present in one of the seven photosynthesis-temperature curves we measured, *Z. muelleri* in summer at Moreton Bay. Photosynthesis-temperature curves for seagrass are expected to have a negative skew, which indicates, at temperatures below *T*_*opt*_, a gradual increase in photosynthesis rate with temperature, and rapid decrease in photosynthesis rate above *T*_*opt*_. Most of our results were consistent with this expectation. In contrast, positive skew indicates, at temperatures below *T*_*opt*_, a sharp increase in photosynthesis with temperature, and a gradual decrease in photosynthesis rate above *T*_*opt*_. Positive skew appeared to be expressed by our *Z. muelleri* data; as a result, seven of the 12 models fitted the *Z. muelleri* data better (adjusted R^2^ value of 0.65–66) than the Yan and Hunt model (adjusted R^2^ value of 0.55, see Supplementary Table S2). If positive skew of the photosynthesis-temperature curve is a species-specific characteristic of *Z. muelleri*, then models other than the Yan and Hunt model are better suited to modelling the dependence of *Z. muelleri* photosynthesis on temperature. However, for this study only one dataset for the species *Z. muelleri* was collected, whilst three datasets each for the other two species *C. serrulata* and *H. uninervis* were collected. Further measurements of *Z. muelleri* photosynthesis are therefore necessary to confirm whether the positive skew of its photosynthesis-temperature curve is reproducible (and therefore a species-specific trait) or not. If the photosynthesis-temperature curve of this species (or others) consistently does not fit well to the Yan and Hunt model, one of the other investigated models that easily yields biologically-meaningful parameters (i.e. the Briére-1, Briére-2, Deutsch, or van der Heide models) may be more appropriate.

### Comparison with previous model selection approaches

In this paper we chose the best model based on goodness of fit and obtaining biologically-meaningful parameters (Fig. 2). This approach builds upon previous model selection studies which have considered both these criteria, though not in such a systematic manner. For example, model selection of temperature-dependent biological rates in two recent studies was based primarily on (1) mean Akaike weight^34^, and (2) adjusted R^2^ and AIC^17^, although in both studies the selected model was argued to be also advantageous for estimating *T*_min_, *T*_*opt*_ and *T*_max_.

In the two aforementioned studies, the Lactin and van der Heide models were found to be the best^17^,^34^, although we ruled out both these models. In our study, the Lactin and van der Heide models were both in the bottom six of 12 models based on goodness of fit, ranked either by mean Akaike weight or mean Schwarz weight. For the van der Heide model, this reduced fit likely occurred because the predicted temperatures *T*_min_, *T*_*opt*_ and *T*_max_ of this model are not mutually independent, which places significant restrictions on the exact shapes of temperature-dependence that can be captured. For the Lactin model, it is not easy to obtain biologically-meaningful parameters because solution of analytically intractable equations is required to write this model in terms of *T*_max_. In contrast, the Yan and Hunt model has a simple algebraic form in terms of *T*_max_, and does not have interdependence of *T*_min_, *T*_*opt*_ and *T*_max_ values because it assumes *T*_min_ = 0, thereby freeing up its remaining parameters to adequately capture the shape of the temperature-dependence of seagrass photosynthesis.

## Conclusion

Model selection that specifically accounts for both goodness of fit and biologically-meaningful parameters is likely to identify a more useful model than a model selection process that considers only best fit. In particular, ensuring that model parameters are transferable will likely increase usage of these parameters for (1) comparison with experimentally-measurable quantities to provide an indication of system state, and (2) implementation in multiple types of models. In our case, the thermal optima and maxima identified for three tropical seagrass species can be used as upper temperature limits to assess the vulnerability of these species to ocean warming, and can be implemented in future parameterisations of plant growth models that are based on carbon balance. Overall, widespread usage of biologically-meaningful parameters will facilitate greater connections between the work of modellers and ecologists, thus enriching the research of both fields for the future.

## Methods

Photosynthetic rates were measured over a large temperature range for three tropical seagrass species growing in winter and summer at two different latitudes. For each species, season and latitude, the dependence of photosynthesis rate on temperature was fitted to 12 different empirical models. The best photosynthesis-temperature model for tropical seagrass was identified, based on goodness of fit and the ease at which biologically-meaningful parameters can be obtained from the model.

### Study sites

**Figure 4:**
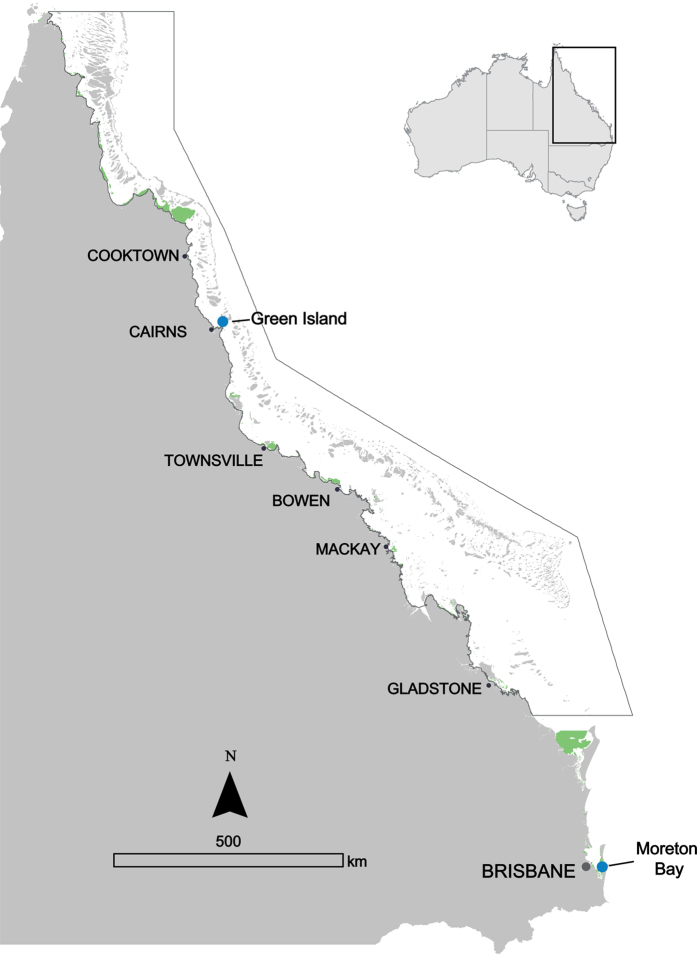
Green Island and Moreton Bay study sites, off the coast of Queensland, Australia. Seagrass distribution (shown in green) is reproduced from McKenzie *et al*.^50^ for Green Island and Roelfsema *et al*.^51^ for Moreton Bay. All seagrass data for this map is publicly available in PANGAEA^52^,^53^. The map was produced using ArcGIS for Desktop version 10.2 (Esri 2013) (http://www.esri.com/software/arcgis/arcgis-for-desktop) and Adobe Illustrator CC 2015 (http://www.adobe.com/au/creativecloud.html).

The study was undertaken at two locations (hereafter called “latitudes”) on the eastern coast of Queensland, Australia: (1) Green Island, near Cairns, in the Great Barrier Reef (16^°^45′17.70′′S, 145°58′22.74′′E), and (2) Moreton Bay, near Brisbane (27^°^29′31.70′′S, 153 °24′4.61′′E). Green Island is a nearshore reef habitat, and has a seagrass community consisting of several tropical species^35^. Moreton Bay is a partially-enclosed embayment, with multiple seagrass species growing in a large shallow area on its eastern side called Eastern Banks^36^. Moreton Bay is approximately 1500 km south of Green Island, as shown in Fig. 4. All data used in this paper were collected from: (1) the northern waters of Green Island and (2) One Mile, a site within Eastern Banks that is adjacent to the north-western coast of North Stradbroke Island, within Moreton Bay.

### Data collection

Whole seagrass shoots were collected from Green Island and Moreton Bay. Photosynthesis was measured within 24 hours of collection. If photosynthesis could not be measured on the same day as collection, intact cores of shoots, rhizomes and sediment were collected and placed into submerged garden pots that were kept overnight within tanks onsite with re-circulated water and gas bubblers at ambient water temperature, and photosynthesis was measured the following morning. For comparison between seasons and latitudes, shoots were collected from Green Island in January 2015 (summer), and from Moreton Bay in February/March 2015 (summer) and June 2015 (winter). For comparison between species, shoots of the seagrass species *H. uninervis* and *C. serrulata* were collected; in addition, shoots of the seagrass species *Z. muelleri* were collected from Moreton Bay in summer. This provided a total of seven different latitude/species/season combinations, hereafter called “treatments”, from which to obtain photosynthesis-temperature curves of seagrass.

Photosynthesis of seagrass leaves was measured using the O_2_ optode method^37^,^38^, specifically by using optical oxygen sensors (“optodes” PreSens, Sensor spots-Pst3) and two PreSens Oxy 4 four-channel fiber-optic oxygen meters that were placed within small incubation chambers. Seagrass leaves were held upright in the chamber to mimic natural orientation. Two arrays of four chambers were run at each time. Each optode was calibrated according to Collier *et al*.^18^ prior to initial measurements. Small transparent acrylic chambers (70 mL) were set into an array of four separate chambers to allow four parallel measures, and temperature was controlled using a flow-through water system connected to a water bath (Lauda, Ecoline RE 106). The temperature bath and temperature loggers were calibrated against a precision NATA certified mercury thermometer. Each chamber was stirred with a magnetic stirrer bar. A blank chamber was included in each array of four chambers to test for blank production.

Dark respiration of seagrass leaves was measured from oxygen consumption in the dark, and net photosynthetic rates were then measured when the same leaf was illuminated at saturating light conditions, at the same temperature. Net photosynthesis rates were measured at the light level of 400 *μ*mol photons m^−2^ s^−1^, which is a saturating light level for all three seagrass species measured^39^,^40^,^41^. Illumination was provided by white LED lamps^42^ and measured using a photosynthethically active radiation probe (MQ-200, Apogee Instruments) that was calibrated against a manufacturer-calibrated 2*π* light sensor (LiCor).

Net photosynthesis and dark respiration rates were measured at seven different temperatures, ranging from 15 to 43 °C in winter and 17 to 43 °C in summer. Six replicates were used for each species. A minimum of 40 minutes was allowed after changing the temperature of the water bath to enable the temperature of the incubation chambers to reach the target temperature. Seawater within the chambers was replaced prior to measurements at the two highest temperatures. Previous tests of the water discarded from chambers showed very small changes in chamber pH (mean ΔpH = 0.05) over the incubation period when using this water changing regime.

After measurements of net photosynthesis and dark respiration were completed, seagrass leaves were rinsed in freshwater and dried for 48 h at 60 °C, to obtain the dry leaf mass and therefore normalise net photosynthesis and dark respiration rates to dry leaf mass. Oxygen (O_2_) consumption rates were then converted to carbon (C) fixation rates by assuming that the amount of carbon fixed/released during photosynthesis and respiration was equal to the amount of O_2_ evolved/fixed respectively^43^,^44^,^45^. Together the conversions yielded net photosynthesis and dark respiration rates in units of g C g^−1^ DW d^−1^. Corresponding values of net photosynthesis and dark respiration rate for each replicate and temperature were subtracted to obtain the temperature-dependent gross photosynthesis rate, in the same units.

### Model fitting and selection

To adequately capture the temperature dependence of seagrass photosynthesis, we first identified suitable models that have been proposed in the literature. Four recent papers^1^,^17^,^34^,^46^ have compared empirical equations for the temperature dependence of biological processes. In total, these four papers considered 28 different models. Starting with these 28 models, we reduced the total number of models examined to 12, by only keeping models that satisfied the following four criteria:

1. *The model provides the correct general shape of the photosynthesis-temperature curve.* Specifically, the model predicts a rise in photosynthesis rate with temperature at low temperatures, up to an optimum *T*_*opt*_, and a decrease in photosynthesis rate at temperatures higher than *T*_*opt*_.
2. *The model is not symmetric with respect to the optimum temperature.* In other words, the model allows the photosynthesis rate at a temperature Δ*T* degrees lower than the optimum *T*_*opt*_, to be different to the photosynthesis rate at a temperature Δ*T* degrees higher than the optimum (i.e. the model allows that P(*T*_*opt*_ −Δ*T*) ≠ P(*T*_*opt*_ + Δ*T*)).
3. *The model has no more than 4 free parameters.* This criterion reduces the chances of model overfitting.
4. *The model can be written unambiguously in terms of the maximum photosynthesis rate P*_max_ *and optimum temperature T*_*opt*_. The model can therefore be used to identify the two parameters that characterise the thermal optimum.

The 12 models that satisfied these four criteria, and the biologically-meaningful parameters that can be obtained from them, are summarised in Table 1 and mathematically defined in Supplementary Section S1. All 12 models were written in terms of *P*_max_ and *T*_*opt*_ (see Criterion 4 above); to accomplish this required some algebraic manipulations, described in Supplementary Section S2. The convention of this paper is to name the models after the first one or two authors who first suggested the model for application to temperature-dependent biological rates.

The 12 models listed in Table 1 were individually fitted to the seven treatments of seagrass gross photosynthesis against temperature using nonlinear regression. The regression was performed using ordinary least-squares fitting via the NonLinearModel.fit command in MATLAB^47^. To compare model fits to the data, four statistical metrics were calculated and compared: adjusted R-squared, refined index of agreement^48^, and Akaike and Schwarz weights^49^. These four metrics are indicators of model performance, and the latter two (Akaike and Schwarz weights) are specifically designed for comparison between different models^2^,^46^. Akaike and Schwarz weights were calculated from the *small sample unbiased* Akaike Information Criterion (AIC_*c*_) and the Bayesian-Schwarz Information Criterion (BIC), respectively. Akaike weights were calculated from the AIC_*c*_ instead of the Akaike Information Criterion (AIC) because the number of model parameters *p* exceeded *n*/40, where *n* is the sample size, for all models and treatments in our study (*p* ≥ 3, *n* = 42)^2^.

For model selection in this paper, the ability of the model fitting to yield biologically-meaningful parameters was considered of similar importance as goodness of fit. Hence, the 12 models were also evaluated by the ease at which biologically-meaningful parameters, particularly *T*_*opt*_, *T*_max_ and *P*_max_, could be obtained from them. We considered prediction of the minimum temperature *T*_min_ to be less important for model selection, because seagrasses are primarily threatened by ocean warming^22^,^23^,^24^,^25^.

Finally, the shape parameter *Q*_10_ was also calculated for each model and treatment combination. *Q*_10_ is the factor increase in biological rate with 10 °C increase in temperature^30^, for temperatures below the optimum. For each of the 12 models fitted to the data, an exponential function of the form


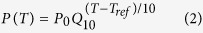


was fitted to the data for temperatures less than the optimum temperature *T*_*opt*_ using nonlinear regression^47^. In equation (2), *P*_0_ is the photosynthesis rate at the reference temperature *T*_*ref*_ = 20 °C, following the convention of Baird *et al*.^7^. Because the value of *T*_*opt*_ depends on which of the 12 models from Table 1 is fitted to the data, we calculated values of *Q*_10_ for each model and treatment combination. Although the exponential rise in photosynthetic rate with temperature will begin to plateau at temperatures slightly less than *T*_*opt*_, the temperature difference between photosynthesis measurements at adjacent temperatures in our study was assumed to be sufficiently large so that the impact of this effect on calculation of *Q*_10_ could be neglected.

## Additional Information

**How to cite this article:** Adams, M. P. *et al*. Model fit versus biological relevance: Evaluating photosynthesis-temperature models for three tropical seagrass species. *Sci. Rep.* **7**, 39930; doi: 10.1038/srep39930 (2017).

**Publisher's note:** Springer Nature remains neutral with regard to jurisdictional claims in published maps and institutional affiliations.

## Supplementary Material

Supplementary Information

## Figures and Tables

**Table 1:** Summary of the 12 models fitted to photosynthesis-temperature curves in this paper.

Model	Minimum temperature	Optimum temperature	Maximum photosynthesis rate	Maximum temperature
Briére-1^54^	*T*_min_	*T*_*opt*_	*P*_max_	*T*_max_
Briére-2^54^	*T*_min_	*T*_*opt*_	*P*_max_	*T*_max_
Deutsch^55^	−∞	*T*_*opt*_	*P*_max_	*T*_max_
Johnson^56^	0 K	*T*_*opt*_	*P*_max_	+∞
Lactin^57^	Undefined or *T*_min_^a^	*T*_*opt*_	*P*_max_	*T*_max_
O’Neill^58^	−∞	*T*_*opt*_	*P*_max_	*T*_max_
Ratkowsky^16^	*T*_min_	*T*_*opt*_	*P*_max_	*T*_max_
Room^59^	−∞	*T*_*opt*_	*P*_max_	+∞
Spain^60^	−∞	*T*_*opt*_	*P*_max_	*T*_max_
Thébault^61^	*T*_min_	*T*_*opt*_	*P*_max_	+∞
van der Heide^17^	*T*_min_	*T*_*opt*_	*P*_max_	*T*_max_
Yan and Hunt^26^	0 °C	*T*_*opt*_	*P*_max_	*T*_max_

These models are mathematically defined in Supplementary Section S1. ^a^The Lactin model may or may not have a *T*_min_, depending on the sign of its intermediate parameter *k*_2_.

**Table 2:** Akaike weights *w*_*A*_ of all models fitted to all photosynthesis-temperature curves.

Species, Season, Location	Briére-1	Briére-2	Deutsch	Johnson	Lactin	O’Neill	Ratkowsky	Room	Spain	Thébault	van der Heide	Yan and Hunt
*C. serrulata*, Summer, Green Island	0.0977	0.0859	0.1030	0.0566	0.0933	0.0880	0.1221	0.1320	0.0075	0.0001	0.0166	0.1974
*C. serrulata*, Summer, Moreton Bay	0.0720	0.0635	0.0819	0.1026	0.1190	0.1551	0.1438	0.0859	0.0827	0.0000	0.0000	0.0937
*C. serrulata*, Winter, Moreton Bay	0.0005	0.0005	0.0030	0.8553	0.0019	0.0541	0.0070	0.0037	0.0011	0.0630	0.0000	0.0100
*H. uninervis*, Summer, Green Island	0.0510	0.1104	0.1310	0.0575	0.0247	0.0531	0.1114	0.0921	0.0054	0.0409	0.2170	0.1056
*H. uninervis*, Summer, Moreton Bay	0.0136	0.0135	0.0808	0.1709	0.0535	0.2602	0.1170	0.0914	0.0188	0.0000	0.0000	0.1804
*H. uninervis*, Winter, Moreton Bay	0.3373	0.1639	0.1126	0.0018	0.0861	0.0193	0.0589	0.1050	0.0067	0.0000	0.0006	0.1079
*Z. muelleri*, Summer, Moreton Bay	0.0000	0.1096	0.1087	0.2172	0.0000	0.0812	0.1103	0.2264	0.0000	0.1405	0.0046	0.0016
Mean	0.0817	0.0782	0.0887	0.2088	0.0541	0.1016	0.0958	0.1052	0.0174	0.0349	0.0341	0.0995
Mean (excluding *C. serrulata*, Winter, Moreton Bay)	0.0953	0.0911	0.1030	0.1011	0.0628	0.1095	0.1106	0.1221	0.0202	0.0302	0.0398	0.1144

**Table 3:** Schwarz weights *w*_*B*_ of all models fitted to all photosynthesis-temperature curves.

Species, Season, Location	Briére-1	Briére-2	Deutsch	Johnson	Lactin	O’Neill	Ratkowsky	Room	Spain	Thébault	van der Heide	Yan and Hunt
*C. serrulata*, Summer, Green Island	0.1451	0.0670	0.0804	0.0442	0.0728	0.0686	0.0952	0.1030	0.0058	0.0000	0.0246	0.2932
*C. serrulata*, Summer, Moreton Bay	0.1193	0.0552	0.0712	0.0892	0.1035	0.1349	0.1250	0.0747	0.0719	0.0000	0.0000	0.1551
*C. serrulata*, Winter, Moreton Bay	0.0009	0.0005	0.0029	0.8472	0.0018	0.0536	0.0069	0.0037	0.0011	0.0624	0.0000	0.0189
*H. uninervis*, Summer, Green Island	0.0726	0.0825	0.0979	0.0430	0.0185	0.0397	0.0832	0.0688	0.0040	0.0306	0.3089	0.1502
*H. uninervis*, Summer, Moreton Bay	0.0220	0.0115	0.0688	0.1454	0.0455	0.2214	0.0995	0.0777	0.0160	0.0000	0.0000	0.2922
*H. uninervis*, Winter, Moreton Bay	0.4578	0.1168	0.0802	0.0013	0.0613	0.0138	0.0420	0.0748	0.0048	0.0000	0.0008	0.1465
*Z. muelleri*, Summer, Moreton Bay	0.0000	0.1090	0.1082	0.2162	0.0000	0.0809	0.1098	0.2253	0.0000	0.1398	0.0080	0.0028
Mean	0.1168	0.0632	0.0728	0.1981	0.0434	0.0875	0.0802	0.0897	0.0148	0.0333	0.0489	0.1513
Mean (excluding *C. serrulata*, Winter, Moreton Bay)	0.1361	0.0737	0.0844	0.0899	0.0503	0.0932	0.0925	0.1041	0.0171	0.0284	0.0571	0.1733

**Table 4:** Parameters (mean ± SE) of all photosynthesis-temperature curves of tropical seagrass, found using the Yan and Hunt model.

Species	Season	Location	*P*_max_ (mg C g^−1^ DW h^−1^)	*T*_*opt*_ (°C)	*T*_max_ (°C)	*P*_0_ (mg C g^−1^ DW h^−1^)	*Q*_10_	Adj. R^2^
*C. serrulata*	Summer	Green Island	3.9 ± 0.2	34.9 ± 0.5	43.7 ± 0.3	1.3 ± 0.2	2.4 ± 0.3	0.76
*C. serrulata*	Summer	Moreton Bay	3.9 ± 0.1	35.4 ± 0.3	44.2 ± 0.3	1.3 ± 0.1	2.2 ± 0.2	0.84
*C. serrulata*	Winter	Moreton Bay	2.9 ± 0.1	35.8 ± 0.3	44.7 ± 0.3	0.8 ± 0.1	2.7 ± 0.2	0.84
*H. uninervis*	Summer	Green Island	3.9 ± 0.3	34.0 ± 0.9	44.6 ± 0.9	1.5 ± 0.3	2.7 ± 0.7	0.44
*H. uninervis*	Summer	Moreton Bay	5.2 ± 0.1	35.8 ± 0.2	44.4 ± 0.2	1.6 ± 0.1	2.4 ± 0.2	0.90
*H. uninervis*	Winter	Moreton Bay	5.5 ± 0.2	34.9 ± 0.3	44.6 ± 0.3	2.1 ± 0.2	2.1 ± 0.2	0.85
*Z. muelleri*	Summer	Moreton Bay	4.3 ± 0.3	30.9 ± 1.0	43.6 ± 0.7	2.5 ± 0.4	2.2 ± 0.4	0.55
