# Supplementary material for: Model fit versus biological relevance: Evaluating photosynthesis-temperature models for three tropical seagrass species
Source: Sci Rep. 2017 Jan 4;7:39930. doi: 10.1038/srep39930 (PMC5209739; doi:10.1038/srep39930)
Supplement: Supplementary Information [file srep39930-s1.pdf]

Model fit versus biological relevance: Evaluating photosynthesis-temperature models for three tropical seagrass species (Supplementary Information)

Matthew P. Adams[a,*], Catherine J. Collier[b,c], Sven Uthicke[d], Yan X. Ow[b,d,e], Lucas Langlois[c], Katherine R. O'Brien[a]

[a]*School of Chemical Engineering, The University of Queensland, St Lucia, QLD 4072, Australia*
[b]*College of Marine and Environmental Science, James Cook University, Townsville, QLD 4811, Australia*
[c]*Centre for Tropical Water & Aquatic Ecosystem Research (TropWATER), James Cook University, Cairns, QLD 4870, Australia*
[d]*Australian Institute of Marine Science, PMB No. 3, Townsville, QLD 4811, Australia*
[e]*Experimental Marine Ecology Laboratory, Department of Biological Sciences, National University of Singapore, 117557, Singapore*

S1. Mathematical definition of the 12 models

In order of preference, all models are defined in terms of the parameters $P_{\max}$, T_{opt}, $T_{\max}$, $T_{\min}$, and thereafter arbitrary parameters a and b. Any intermediate parameters without physical meaning are defined as k_1 and k_2. For models containing either $T_{\max}$ or $T_{\min}$, $P(T \geq T_{\max}) = 0$ and $P(T \leq T_{\min}) = 0$ is assumed, respectively. Temperature is always written in units of °C. The Johnson, Lactin and Spain models require substantial algebraic manipulation from their original forms to be written in terms of $P_{\max}$ and T_{opt}; derivations for the equations of these models, as used in this paper, are provided in Supplementary Section S2.

S1.1. Briére-1 model

The Briére-1 model (Briére et al., 1999) has three parameters $P_{\max}$, T_{opt} and $T_{\max}$:

$$P(T) = k_1 T \left(T - T_{\min}\right) \sqrt{T_{\max} - T}, \tag{S1.1a}$$

$$k_1 = P_{\max} / \left[T_{opt} \left(T_{opt} - T_{\min}\right) \sqrt{T_{\max} - T_{opt}}\right], \tag{S1.1b}$$

$$T_{\min} = \frac{T_{opt}\left(5T_{opt} - 4T_{\max}\right)}{3T_{opt} - 2T_{\max}}. \tag{S1.1c}$$

S1.2. Briére-2 model

The Briére-2 model (Briére et al., 1999) has four parameters $P_{\max}$, T_{opt}, $T_{\max}$ and $T_{\min}$:

$$P(T) = k_1 T \left(T - T_{\min}\right) \left(T_{\max} - T\right)^{k_2}, \tag{S1.2a}$$

$$k_1 = P_{\max} / \left[T_{opt} \left(T_{opt} - T_{\min}\right) \left(T_{\max} - T_{opt}\right)^{k_2}\right], \tag{S1.2b}$$

$$k_2 = \frac{\left(2T_{opt} - T_{\min}\right)\left(T_{\max} - T_{opt}\right)}{T_{opt}\left(T_{opt} - T_{\min}\right)}. \tag{S1.2c}$$

S1.3. Deutsch model

The Deutsch model (Deutsch et al., 2008) has four parameters $P_{\max}$, T_{opt}, $T_{\max}$ and a:

$$P(T) = \begin{cases} P_{\max} \exp\left[-a\left(T - T_{opt}\right)^2\right], & T \leq T_{opt}, \\ P_{\max}\left[1 - \left(\dfrac{T - T_{opt}}{T_{opt} - T_{\max}}\right)^2\right], & T \geq T_{opt}, \end{cases} \tag{S1.3}$$

*Corresponding author. *Email address*: m.adams5@uq.edu.au (M. P. Adams).

where $a > 0$ is in units of $^{\circ}C^{-1/2}$.

S1.4. Johnson model

The Johnson model (Johnson et al., 1974) has four parameters $P_{\max}$, T_{opt}, a and b:

$$P(T) = \frac{k_1(T + 273.15)\exp\left[-a/(T + 273.15)\right]}{1 + \exp\left[k_2 - b/(T + 273.15)\right]}, \tag{S1.4a}$$

$$k_1 \approx \frac{P_{\max} b}{(b - a)(T_{opt} + 273.15)\exp\left[-a/(T_{opt} + 273.15)\right]}, \tag{S1.4b}$$

$$k_2 \approx \frac{b}{T_{opt} + 273.15} - \ln\left[\frac{b}{a} - 1\right], \tag{S1.4c}$$

where $a > 0$ and $b > 0$ are in units of °C. The derivation of equations (S1.4a)-(S1.4c) from Johnson et al. (1974) is provided in Supplementary Section S2.1.

S1.5. Lactin model

The Lactin model (Lactin et al., 1995) has four parameters $P_{\max}$, T_{opt}, $T_{\max}$ and a:

$$P(T) = \exp(aT) - k_1 \exp\left(a\left[T_{opt} - (T_{opt} - T)/k_1\right]\right) + k_2, \tag{S1.5a}$$

$$k_1 = 1 - (P_{\max} - k_2)\exp(-aT_{opt}), \tag{S1.5b}$$

$$0 = \exp(aT_{\max}) - k_1 \exp\left(a\left[T_{opt} - (T_{opt} - T_{\max})/k_1\right]\right) + k_2, \tag{S1.5c}$$

where $a > 0$ is in units of $^{\circ}C^{-1}$. Equation (S1.5c) is analytically intractable, and requires a numerical root-finding method to solve for k_2. The positive or negative sign of k_2 determines whether the Lactin model predicts an undefined or finite minimum temperature, respectively. The derivation of equations (S1.5a)-(S1.5c) from Lactin et al. (1995) is provided in Supplementary Section S2.2.

S1.6. O'Neill model

The O'Neill model (O'Neill et al., 1972) has four parameters $P_{\max}$, T_{opt}, $T_{\max}$ and a:

$$P(T) = P_{\max}\left(\frac{T_{\max} - T}{T_{\max} - T_{opt}}\right)^a \exp\left[a\left(1 - \frac{T_{\max} - T}{T_{\max} - T_{opt}}\right)\right], \tag{S1.6}$$

where $a > 0$ has no units. Empirical equations for the dependence of a on Q_{10} have been formulated (e.g. Li & Yakupitiyage (2003)) although these equations are specific to the O'Neill model and cannot be used for other models.

S1.7. Ratkowsky model

The Ratkowsky model (Ratkowsky et al., 1983) has four parameters $P_{\max}$, T_{opt}, $T_{\max}$ and $T_{\min}$:

$$P(T) = k_1\left[(T - T_{\min})\left(1 - e^{k_2(T - T_{\max})}\right)\right]^2, \tag{S1.7a}$$

$$k_1 = P_{\max}/\left[(T_{opt} - T_{\min})\left(1 - e^{k_2(T_{opt} - T_{\max})}\right)\right]^2, \tag{S1.7b}$$

$$0 = 1 - \left[1 + k_2(T_{opt} - T_{\min})\right]e^{k_2(T_{opt} - T_{\max})}. \tag{S1.7c}$$

Equation (S1.7c) is analytically intractable, and requires a numerical root-finding method to solve for k_2.

S1.8. Room model

The Room model (Room, 1986) has four parameters $P_{\max}$, T_{opt}, a and b:

$$P(T) = \begin{cases} P_{\max} \exp\left[-a\left(T - T_{opt}\right)^2\right], & T \leq T_{opt}, \\ \\ P_{\max} \exp\left[-b\left(T - T_{opt}\right)^2\right], & T \geq T_{opt}, \end{cases} \tag{S1.8}$$

where $a > 0$ and $b > 0$ are in units of $^\circ\mathrm{C}^{-1/2}$.

S1.9. Spain model

The Spain model (Spain, 1982) has four parameters $P_{\max}$, T_{opt}, $T_{\max}$ and a:

$$P(T) = k_1 e^{aT/(T_{\max} - T_{opt})}\left(1 - e^{-k_2(T_{\max} - T)}\right), \tag{S1.9a}$$

$$k_1 = P_{\max} / \left[e^{aT_{opt}/(T_{\max} - T_{opt})}\left(1 - e^{-k_2(T_{\max} - T_{opt})}\right)\right], \tag{S1.9b}$$

$$0 = a - (a + k_2(T_{\max} - T_{opt}))e^{-k_2(T_{\max} - T_{opt})}, \tag{S1.9c}$$

where a has no units and must satisfy $0 < a < 1$. Equation (S1.9c) is analytically intractable, and requires a numerical root-finding method to solve for k_2. An empirical equation for the dependence of a on Q_{10} has been formulated (Spain, 1982) although this equation is specific to the Spain model and cannot be used for other models. The derivation of equations (S1.9a)-(S1.9c) from Spain (1982) and the derivation of inequality $0 < a < 1$ is provided in Supplementary Section S2.3.

S1.10. Thébault model

The Thébault model (Thébault, 1985) has four parameters $P_{\max}$, T_{opt}, $T_{\min}$ and a:

$$P(T) = P_{\max} \frac{2ak_1}{k_1^2 + 2(a-1)k_1 + 1}, \tag{S1.10a}$$

$$k_1 = \frac{T - T_{\min}}{T_{opt} - T_{\min}}, \tag{S1.10b}$$

where $a > 0$ has no units. In its original form, the model of Thébault (1985) contained the parameter β instead of a, where $a = 1 + \beta$. We defined $a = 1 + \beta$ because the parameter constraint $a > 0$ is more convenient than $\beta > -1$.

S1.11. van der Heide model

The van der Heide model (van der Heide et al., 2006) has three parameters $P_{\max}$, T_{opt} and $T_{\max}$:

$$P(T) = k_1 T\left(T - T_{\min}\right)\left(T_{\max} - T\right), \tag{S1.11a}$$

$$k_1 = P_{\max} / \left[T_{opt}\left(T_{opt} - T_{\min}\right)\left(T_{\max} - T_{opt}\right)\right], \tag{S1.11b}$$

$$T_{\min} = \frac{T_{opt}\left(3T_{opt} - 2T_{\max}\right)}{2T_{opt} - T_{\max}}. \tag{S1.11c}$$

S1.12. Yan and Hunt model

The Yan and Hunt model (Yan & Hunt, 1999) has three parameters $P_{\max}$, T_{opt}, and $T_{\max}$:

$$P(T) = P_{\max}\left(\frac{T_{\max} - T}{T_{\max} - T_{opt}}\right)\left(\frac{T}{T_{opt}}\right)^{T_{opt}/(T_{\max} - T_{opt})}. \tag{S1.12}$$

S2. Mathematical derivations

S2.1. Derivation of the Johnson model

The Johnson model, defined in equations (S1.4a)-(S1.4c), differs from its original form (Johnson et al., 1974). Here, we provide the derivation of these equations.

The original model of Johnson et al. (1974) considers an enzyme reaction at rate I given by

$$I = \frac{cT \exp\left[-\Delta H^{\ddagger}/(RT)\right]}{1 + \exp\left[-\Delta H_1/(RT)\right] \exp\left[\Delta S_1/R\right]}, \tag{S2.1}$$

where c is a proportionality constant, T is the temperature in Kelvin, $\Delta H^{\ddagger}$ is approximately equal to the activation energy, R is the universal gas constant, and ΔH_1 and ΔS_1 are the increase in enthalpy and entropy of the reaction. Substituting $I = P$, $c = k_1$, $\Delta H^{\ddagger}/R = a$, $\Delta H_1/R = b$ and $\Delta S_1/R = k_2$ into equation (S2.1), and rewriting temperature in units of °C, yields equation (S1.4a),

$$P(T) = \frac{k_1(T + 273.15) \exp\left[-a/(T + 273.15)\right]}{1 + \exp\left[k_2 - b/(T + 273.15)\right]}.$$

To obtain analytical expressions for parameters k_1 and k_2 in terms of $P_{\max}$ and T_{opt}, we temporarily approximate the factor $k_1(T + 273.15)$ by a constant C, which is an appropriate assumption over the temperature range considered in this paper (15-40°C),

$$P(T) \approx \frac{C \exp\left[-a/(T + 273.15)\right]}{1 + \exp\left[k_2 - b/(T + 273.15)\right]}. \tag{S2.2}$$

With some algebraic manipulation, equation (S1.4c) can be obtained from equation (S2.2) by setting $\left.\frac{\mathrm{d}P}{\mathrm{d}T}\right|_{T=T_{opt}} = 0$. Subsequent substitution of equation (S1.4c) and $P(T_{opt}) = P_{\max}$ into equation (S1.4a) gives equation (S1.4b).

S2.2. Derivation of the Lactin model

The Lactin model, defined in equations (S1.5a)-(S1.5c), differs from its original form (Lactin et al., 1995). Here, we provide the derivation of these equations.

The original model of Lactin et al. (1995) considers a biological rate r given by

$$r = \exp(\rho T) - \exp\left([\rho T_{\max} - (T_{\max} - T)/\Delta]\right) + \lambda \tag{S2.3}$$

where ρ, Δ and λ are fitted parameters, and $T_{\max}$ indicates the maximum temperature only if $\lambda = 0$. To obtain a definition of $T_{\max}$ that does not require $\lambda = 0$, we replace $T_{\max}$ by T_*, and substitute $r = P$, $\rho = a$, $\Delta = k_1/a$ and $\lambda = k_2$ to obtain:

$$P(T) = \exp(aT) - \exp\left(a\left[T_* - (T_* - T)/k_1\right]\right) + k_2 \tag{S2.4}$$

Setting $\left.\frac{\mathrm{d}P}{\mathrm{d}T}\right|_{T=T_{opt}} = 0$, we can obtain from equation (S2.4) that

$$T_* = T_{opt} + \frac{k_1 \ln(k_1)}{a(k_1 - 1)}. \tag{S2.5}$$

Substitution of equation (S2.5) into equation (S2.4) gives equation (S1.5a). Subsequent substitution of $P(T_{opt}) = P_{\max}$ into equation (S1.5a) gives equation (S1.5b). Finally, substitution of $P(T_{\max}) = 0$ into equation (S1.5a) gives equation (S1.5c).

S2.3. Derivation of the Spain model

The Spain model, defined in equations (S1.9a)-(S1.9c), differs from its original form in Spain (1982). Here, we provide the derivation of these equations, as well as explain why the parameter a of this model must satisfy the inequality $0 < a < 1$.

The original model of Spain (1982) considers a biological rate k_T given by

$$k_T = k_0 e^{aT} \left(1 - b e^{cT}\right), \tag{S2.6a}$$

$$b = e^{-cT_{\max}}, \tag{S2.6b}$$

$$c = \ln\left(\frac{a}{a+c}\right) / \left(T_{opt} - T_{\max}\right), \tag{S2.6c}$$

where $a > 0$ and $c > 0$ are fitted parameters. Replacing a by $a/(T_{\max} - T_{opt})$ for convenience in subsequent algebra, substituting $k_T = P$, $k_0 = k_1$ and $c = k_2$, and combining equations (S2.6a) and (S2.6b) through the common parameter b, the Spain model can be rewritten as

$$P(T) = k_1 e^{aT/(T_{\max}-T_{opt})} \left(1 - e^{-k_2(T_{\max}-T)}\right), \tag{S2.7a}$$

$$k_2 = \ln\left(\frac{a}{a + k_2(T_{\max} - T_{opt})}\right) / (T_{opt} - T_{\max}). \tag{S2.7b}$$

Equation (S2.7a) is identical to equation (S1.9a). Rearrangement of equation (S2.7b) yields equation (S1.9c). Substitution of $P(T_{opt}) = P_{\max}$ into equation (S1.9a) gives equation (S1.9b).

We next show that $0 < a < 1$. The first part of this inequality, $a > 0$, follows immediately from its definition in the original model of Spain (1982). To show $a < 1$, we introduce

$$\Delta T = T_{\max} - T_{opt}. \tag{S2.8}$$

Because ΔT physically represents the difference between optimum and maximum temperatures, $\Delta T > 0$. Substituting equation (S2.8) into equation (S1.9c) gives

$$0 = a - (a + k_2 \Delta T)\, e^{-k_2 \Delta T}. \tag{S2.9}$$

Since $c = k_2$, and $c > 0$ according to its definition in the original model of Spain (1982), $k_2 > 0$. To find values of k_2 that satisfy equation (S2.9), we define the function $f(k_2)$,

$$f(k_2) = a - (a + k_2 \Delta T) e^{-k_2 \Delta T}, \tag{S2.10}$$

and seek to identify values of $k_2 > 0$ that make $f(k_2) = 0$, subject to the constraints $a > 0$ and $\Delta T > 0$.

When $k_2 = 0$, $f(k_2) = 0$. When $k_2 \to +\infty$, $f(k_2) = a$. As shown in equation (S2.10), the function $f(k_2)$ depends on k_2 only through the summation of two terms that both depend on k_2: the first term $-a e^{-k_2 \Delta T}$ always increases and the second term $-k_2 \Delta T e^{-k_2 \Delta T}$ always decreases. These two competing processes completely define the shape of the function $f(k_2)$. Combining these considerations, the only way that $f(k_2) = 0$ can occur for a value of $k_2 > 0$ is if $f(k_2)$ decreases to negative values at low $k_2 > 0$ (second term dominates at low k_2), then at higher k_2 values the function $f(k_2)$ increases and becomes positive as it approaches a (first term dominates at high k_2), as shown in Supplementary Figure S1.

For the function $f(k_2)$ to demonstrate the behaviour shown in Supplementary Figure S1, the gradient of $f(k_2)$ must be negative at $k_2 = 0$. Substitution of this condition into equation (S2.10) gives

$$\left.\frac{\mathrm{d}f}{\mathrm{d}k_2}\right|_{k_2=0} = (a-1)\Delta T < 0. \tag{S2.11}$$

Since $\Delta T > 0$ from its physical definition, we obtain from equation (S2.11) that $a < 1$, which is the second part of the required inequality $0 < a < 1$.

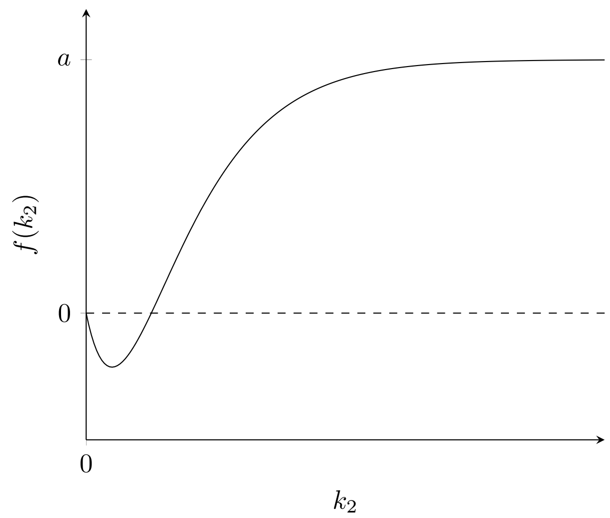

Figure S1: Shape of the function $f(k_2)$ required to obtain $f(k_2 > 0) = 0$. Function $f(k_2)$ is defined in equation (S2.10).

S3. Supplementary Tables

Table S1: Parameters (mean ± SE) for all models and datasets. The units of parameters a and b are model-dependent, and are specified in Supplementary Section S1. The table does not include parameters assumed by certain models (e.g. $T_{\mathrm{min}} = 0\,\mathrm{K}$ in the Johnson model, $T_{\mathrm{min}} = 0°\mathrm{C}$ in the Yan and Hunt model, see Table 1) or redundant parameters whose values can be calculated from other parameters (e.g. T_{min} for the Briére-1, Briére-2 and van der Heide models, see Supplementary Section S1). [a]The O'Neill model was constrained by $T_{\mathrm{max}} \leq 100°\mathrm{C}$ in order to achieve successful model fitting; in this case the model fitting selected the maximum value of T_{max}. [b]The Thébault model was constrained by $T_{\mathrm{min}} \geq 0\,\mathrm{K}$ in order to achieve successful model fitting; in this case the model fitting selected the minimum value of T_{min}. [c]The Spain model parameter a was constrained by $0 < a < 1$; in this case the model fitting selected the maximum value of $a \to 1$.

Species, Season, Location	Model	P_{max} (mg C g^{-1} DW h^{-1})	T_{opt} (°C)	T_{max} (°C)	T_{min} (°C)	a	b	P_0 (mg C g^{-1} DW h^{-1})	Q_{10}
C. serrulata, Summer, Green Island	Briére-1	3.9±0.2	36±0.3	43.1±0.1	-	-	-	1.3±0.2	2.4±0.3
	Briére-2	3.9±0.2	34.9±1	43.3±0.3	14.5±1.9	-	-	1.3±0.2	2.4±0.3
	Deutsch	4±0.2	35±1.2	43.9±0.5	-	5.65E-03±1.48E-03	-	1.3±0.2	2.4±0.3
	Johnson	4.3±0.3	34.9±0.6	-	-	8.34E+03±1.41E+03	4.98E+04±9.37E+03	1.3±0.2	2.4±0.3
	Lactin	3.9±0.2	35.3±0.5	43.6±0.3	-	8.08E-02±5.34E-01	-	1.3±0.2	2.4±0.3
	O'Neill	4.1±0.2	34.6±0.7	46±2.5	-	2.80E+00±1.59E+00	-	1.3±0.2	2.4±0.3
	Ratkowsky	4±0.2	34.7±0.8	44.9±0.7	8.7±3.3	-	-	1.3±0.2	2.4±0.3
	Room	4.1±0.2	35.7±0.8	-	-	5.22E-03±1.10E-03	3.21E-02±1.23E-02	1.3±0.2	2.4±0.3
	Spain	3.9±0.4	36±1.2	43.4±1	-	1±0[c]	-	1.3±0.2	2.4±0.3
	Thébault	4.2±0.3	33.2±0.6	-	-273.2±0[b]	3.11E-04±7.45E-05	-	1.3±0.2	2.5±0.4
	van der Heide	3.8±0.2	33.2±0.4	44.4±0.5	-	-	-	1.3±0.2	2.5±0.4
	Yan and Hunt	3.9±0.2	34.9±0.5	43.7±0.3	-	-	-	1.3±0.2	2.4±0.3
C. serrulata, Summer, Moreton Bay	Briére-1	3.8±0.1	36.2±0.2	43.3±0.1	-	-	-	1.3±0.1	2.2±0.2
	Briére-2	3.9±0.2	37.1±1.1	43.1±0.1	11±2.7	-	-	1.3±0.1	2.2±0.2
	Deutsch	3.9±0.1	37.1±1.1	44.1±0.3	-	4.14E-03±8.00E-04	-	1.3±0.1	2.2±0.2
	Johnson	4.1±0.2	35.7±0.5	-	-	7.57E+03±9.27E+02	4.83E+04±6.00E+03	1.3±0.1	2.2±0.2
	Lactin	3.9±0.1	36.4±0.6	43.8±0.2	-	5.61E-02±5.92E-03	-	1.3±0.1	2.2±0.2
	O'Neill	4±0.1	35.9±0.7	44.7±1.1	-	1.54E+00±6.27E-01	-	1.3±0.1	2.2±0.2
	Ratkowsky	3.9±0.1	36.2±0.7	45±0.5	4.4±2.9	-	-	1.3±0.1	2.2±0.2
	Room	3.9±0.1	37.2±0.8	-	-	4.13E-03±7.00E-04	3.72E-02±1.23E-02	1.3±0.1	2.2±0.2
	Spain	4±0.2	36.7±0.7	43.8±1.2	-	1±0[c]	-	1.3±0.1	2.2±0.2
	Thébault	4±0.2	33.8±0.4	-	-273.2±0[b]	3.51E-04±6.37E-05	-	1.2±0.1	2.6±0.3
	van der Heide	3.6±0.1	33.6±0.3	45.4±0.5	-	-	-	1.2±0.1	2.6±0.3
	Yan and Hunt	3.9±0.1	35.4±0.3	44.2±0.3	-	-	-	1.3±0.1	2.2±0.2
C. serrulata, Winter, Moreton Bay	Briére-1	2.8±0.1	36.3±0.2	43.6±0.2	-	-	-	0.8±0.1	2.7±0.2
	Briére-2	2.8±0.1	37.4±1.2	43.2±0.3	10.4±2.7	-	-	0.8±0.1	2.7±0.2
	Deutsch	2.9±0.1	36.4±1.1	45±0.5	-	4.95E-03±9.79E-04	-	0.8±0.1	2.7±0.2
	Johnson	3.1±0.1	35.1±0.5	-	-	9.62E+03±1.10E+03	3.73E+04±3.83E+03	0.8±0.1	2.7±0.2
	Lactin	2.9±0.1	36.8±0.7	44.3±0.4	-	4.74E-02±4.80E-03	-	0.8±0.1	2.7±0.2
	O'Neill	2.9±0.1	35.6±0.6	47.9±3	-	2.95E+00±1.62E+00	-	0.8±0.1	2.7±0.2
	Ratkowsky	2.9±0.1	36.2±0.7	45.9±0.7	5.7±2.7	-	-	0.8±0.1	2.7±0.2
	Room	2.9±0.1	36.4±0.9	-	-	5.00E-03±8.89E-04	2.07E-02±6.67E-03	0.8±0.1	2.7±0.2
	Spain	2.8±0.4	36.8±0.9	44.3±0.5	-	1±0[c]	-	0.8±0.1	2.7±0.2
	Thébault	3.1±0.1	34.2±0.3	-	-273.2±0[b]	3.20E-04±4.26E-05	-	0.8±0.1	2.7±0.2
	van der Heide	2.6±0.1	34.2±0.5	46.8±0.8	-	-	-	0.8±0.1	2.7±0.2
	Yan and Hunt	2.9±0.1	35.8±0.3	44.7±0.3	-	-	-	0.8±0.1	2.7±0.2
H. uninervis, Summer, Green Island	Briére-1	3.8±0.3	35.6±0.6	43.4±0.3	-	-	-	1.8±0.3	1.8±0.3
	Briére-2	3.9±0.3	32.6±1.6	45.8±3	14.6±2.4	-	-	1.5±0.3	2.7±0.7
	Deutsch	3.9±0.3	30.8±2.4	46.1±1.8	-	9.26E-03±5.47E-03	-	1.5±0.3	2.7±0.7
	Johnson	4±0.4	31.1±1.5	-	-	1.43E+04±6.41E+03	2.63E+04±3.99E+03	1.5±0.3	2.7±0.7
	Lactin	3.9±0.4	35.1±2	44.2±1.2	-	7.62E-02±1.27E+00	-	1.8±0.3	1.8±0.3
	O'Neill	4±0.3	32.1±0.8	100±0[a]	-	7.13E+01±1.66E+01	-	1.5±0.3	2.7±0.7
	Ratkowsky	3.9±0.3	32.2±1.5	48.6±3.2	11.8±4.1	-	-	1.5±0.3	2.7±0.7
	Room	4±0.3	31.4±2.3	-	-	8.20E-03±4.44E-03	7.08E-03±4.27E-03	1.5±0.3	2.7±0.7

	Spain	3.8±0.6	35.3±1	43.9±0.1	-	1±0[c]	-	1.8±0.3	1.8±0.3
	Thébault	4.1±0.4	30.8±1.3	-	-1±32.3	4.41E-02±9.79E-02	-	1.5±0.3	2.7±0.7
	van der Heide	3.9±0.3	33±0.6	45±0.9	-	-	-	1.5±0.3	2.7±0.7
	Yan and Hunt	3.9±0.3	34±0.9	44.6±0.9	-	-	-	1.5±0.3	2.7±0.7
H. uninervis, Summer, Moreton Bay	Briére-1	5±0.1	36.4±0.1	43.4±0.1	-	-	-	1.6±0.1	2.4±0.2
	Briére-2	5.1±0.2	37.2±0.8	43.2±0.2	12.4±1.8	-	-	1.6±0.1	2.4±0.2
	Deutsch	5.1±0.1	36.7±0.8	44.5±0.3	-	4.87E-03±7.30E-04	-	1.6±0.1	2.4±0.2
	Johnson	5.4±0.2	35.5±0.4	-	-	8.60E+03±8.50E+02	4.25E+04±3.97E+03	1.6±0.1	2.4±0.2
	Lactin	5.2±0.2	36.5±0.5	44.1±0.2	-	6.61E-02±7.08E-03	-	1.6±0.1	2.4±0.2
	O'Neill	5.2±0.1	35.8±0.5	46.2±1.5	-	2.21E+00±8.31E-01	-	1.6±0.1	2.4±0.2
	Ratkowsky	5.2±0.2	36.2±0.5	45.4±0.4	6.7±2.1	-	-	1.6±0.1	2.4±0.2
	Room	5.2±0.1	36.8±0.6	-	-	4.83E-03±6.43E-04	2.83E-02±6.88E-03	1.6±0.1	2.4±0.2
	Spain	5.2±0.2	37±0.3	44±0.2	-	1±0[c]	-	1.6±0.1	2.4±0.2
	Thébault	5.3±0.2	34.2±0.3	-	-273.2±0[b]	3.41E-04±4.78E-05	-	1.6±0.1	2.4±0.2
	van der Heide	4.7±0.2	34±0.3	45.9±0.5	-	-	-	1.6±0.1	2.4±0.2
	Yan and Hunt	5.2±0.1	35.8±0.2	44.4±0.2	-	-	-	1.6±0.1	2.4±0.2
H. uninervis, Winter, Moreton Bay	Briére-1	5.4±0.2	35.9±0.2	43.4±0.1	-	-	-	2.1±0.2	2.1±0.2
	Briére-2	5.4±0.2	36±0.9	43.3±0.3	10.4±2.1	-	-	2.1±0.2	2.1±0.2
	Deutsch	5.5±0.2	36.5±1	44.5±0.4	-	3.79E-03±6.78E-04	-	2.1±0.2	2.1±0.2
	Johnson	5.8±0.3	35.4±0.6	-	-	7.03E+03±9.42E+02	4.25E+04±5.91E+03	2.1±0.2	2.1±0.2
	Lactin	5.5±0.2	35.7±0.8	44.2±0.4	-	8.32E-02±1.85E-01	-	2.1±0.2	2.1±0.2
	O'Neill	5.6±0.2	35.3±0.7	45.8±1.8	-	1.88E+00±8.63E-01	-	2.1±0.2	2.1±0.2
	Ratkowsky	5.5±0.2	35.4±0.7	45.8±0.6	4±2.8	-	-	2.1±0.2	2.1±0.2
	Room	5.5±0.2	36.8±0.8	-	-	3.67E-03±5.92E-04	2.86E-02±8.93E-03	2.1±0.2	2.1±0.2
	Spain	5.6±0.3	36.4±0.5	44±0.6	-	1±0[c]	-	2.1±0.2	2.1±0.2
	Thébault	5.5±0.3	33.2±0.5	-	-273.2±0[b]	4.77E-04±8.95E-05	-	2.1±0.2	2.1±0.2
	van der Heide	5.2±0.2	33.4±0.3	45.8±0.5	-	-	-	2.1±0.2	2.1±0.2
	Yan and Hunt	5.5±0.2	34.9±0.3	44.6±0.3	-	-	-	2.1±0.2	2.1±0.2
Z. muelleri, Summer, Moreton Bay	Briére-1	4±0.3	32.7±1.4	43.1±0.2	-	-	-	2.5±0.4	2.2±0.4
	Briére-2	4.7±0.3	28.4±1	47.4±4.2	14.8±1.2	-	-	2.3±0.4	3.5±1.2
	Deutsch	4.7±0.3	27±1.6	44.2±1	-	1.35E-02±6.61E-03	-	2.3±0.4	3.5±1.2
	Johnson	5.1±0.3	28.3±0.9	-	-	1.52E+04±4.56E+03	2.98E+04±3.05E+03	2.3±0.4	3.5±1.2
	Lactin	4.1±0.4	32.3±1.1	43.2±0.6	-	7.17E-02±1.73E+00	-	2.5±0.4	2.2±0.4
	O'Neill	4.9±0.3	29.2±0.6	100±0[a]	-	9.07E+01±1.52E+01	-	2.3±0.4	3.5±1.2
	Ratkowsky	4.8±1.7	28.7±125.9	46.3±1.3	11.6±1.3	-	-	2.3±0.4	3.5±1.2
	Room	5±0.3	28.2±1.4	-	-	1.09E-02±4.38E-03	7.81E-03±2.84E-03	2.3±0.4	3.5±1.2
	Spain	4±0.8	31.2±1.4	42.1±0	-	1±0[c]	-	2.5±0.4	2.2±0.4
	Thébault	5.3±0.4	28.5±0.8	-	-83.7±328.6	2.35E-03±1.38E-02	-	2.3±0.4	3.5±1.2
	van der Heide	4.5±0.3	30.9±0.6	43.5±0.7	-	-	-	2.5±0.4	2.2±0.4
	Yan and Hunt	4.3±0.3	30.9±1	43.6±0.7	-	-	-	2.5±0.4	2.2±0.4

Table 2: Statistical metrics for the goodness of fit between all models and datasets.

Species, Season, Location	Model	Adj. R^2	d_r	AIC_c	w_A	BIC	w_B
C. serrulata, Summer, Green Island	Briére-1	0.75	0.78	93.16	0.1	97.74	0.15
	Briére-2	0.75	0.79	93.41	0.09	99.28	0.07
	Deutsch	0.76	0.78	93.05	0.1	98.92	0.08
	Johnson	0.75	0.77	94.25	0.06	100.12	0.04
	Lactin	0.75	0.78	93.25	0.09	99.12	0.07
	O'Neill	0.75	0.78	93.37	0.09	99.23	0.07
	Ratkowsky	0.76	0.78	92.71	0.12	98.58	0.1
	Room	0.76	0.78	92.55	0.13	98.42	0.1
	Spain	0.72	0.76	98.3	0.01	104.16	0.01
	Thébault	0.67	0.73	107.98	0	113.85	0
	van der Heide	0.73	0.77	96.7	0.02	101.29	0.02
	Yan and Hunt	0.76	0.78	91.75	0.2	96.33	0.29
C. serrulata, Summer, Moreton Bay	Briére-1	0.84	0.81	61.48	0.07	66.07	0.12
	Briére-2	0.84	0.82	61.74	0.06	67.61	0.06
	Deutsch	0.85	0.82	61.23	0.08	67.1	0.07
	Johnson	0.85	0.82	60.78	0.1	66.65	0.09
	Lactin	0.85	0.82	60.48	0.12	66.35	0.1
	O'Neill	0.85	0.82	59.95	0.16	65.82	0.13
	Ratkowsky	0.85	0.82	60.1	0.14	65.97	0.13
	Room	0.85	0.82	61.13	0.09	67	0.07
	Spain	0.85	0.82	61.21	0.08	67.08	0.07
	Thébault	0.76	0.78	81.69	0	87.56	0
	van der Heide	0.76	0.78	79.99	0	84.57	0
	Yan and Hunt	0.84	0.82	60.96	0.09	65.54	0.16
C. serrulata, Winter, Moreton Bay	Briére-1	0.81	0.79	42.18	0	46.76	0
	Briére-2	0.81	0.8	42.16	0	48.03	0
	Deutsch	0.83	0.81	38.55	0	44.42	0
	Johnson	0.87	0.83	27.23	0.86	33.1	0.85
	Lactin	0.83	0.8	39.5	0	45.37	0
	O'Neill	0.85	0.82	32.75	0.05	38.62	0.05
	Ratkowsky	0.84	0.81	36.85	0.01	42.72	0.01
	Room	0.83	0.81	38.11	0	43.98	0
	Spain	0.82	0.81	40.57	0	46.44	0
	Thébault	0.86	0.83	32.45	0.06	38.32	0.06
	van der Heide	0.73	0.76	57.43	0	62.01	0
	Yan and Hunt	0.84	0.81	36.12	0.01	40.71	0.02
H. uninervis, Summer, Green Island	Briére-1	0.42	0.65	138.38	0.05	142.96	0.07

	Briére-2	0.45	0.66	136.84	0.11	142.71	0.08
	Deutsch	0.45	0.67	136.5	0.13	142.37	0.1
	Johnson	0.43	0.66	138.14	0.06	144.01	0.04
	Lactin	0.41	0.65	139.83	0.02	145.7	0.02
	O'Neill	0.46	0.66	138.3	0.05	144.17	0.04
	Ratkowsky	0.45	0.66	136.82	0.11	142.69	0.08
	Room	0.44	0.66	137.2	0.09	143.07	0.07
	Spain	0.39	0.63	142.89	0.01	148.76	0
	Thébault	0.42	0.66	138.82	0.04	144.69	0.03
	van der Heide	0.46	0.66	135.49	0.22	140.07	0.31
	Yan and Hunt	0.44	0.65	136.93	0.11	141.51	0.15
H. uninervis, Summer, Moreton Bay	Briére-1	0.89	0.85	68.05	0.01	72.63	0.02
	Briére-2	0.89	0.85	68.06	0.01	73.93	0.01
	Deutsch	0.9	0.86	64.49	0.08	70.36	0.07
	Johnson	0.9	0.87	62.99	0.17	68.86	0.15
	Lactin	0.9	0.86	65.31	0.05	71.18	0.05
	O'Neill	0.9	0.87	62.15	0.26	68.02	0.22
	Ratkowsky	0.9	0.86	63.75	0.12	69.62	0.1
	Room	0.9	0.86	64.24	0.09	70.11	0.08
	Spain	0.89	0.86	67.4	0.02	73.27	0.02
	Thébault	0.85	0.83	83.41	0	89.28	0
	van der Heide	0.81	0.79	91.32	0	95.9	0
	Yan and Hunt	0.9	0.86	62.88	0.18	67.46	0.29
H. uninervis, Winter, Moreton Bay	Briére-1	0.86	0.83	85.57	0.34	90.15	0.46
	Briére-2	0.85	0.83	87.01	0.16	92.88	0.12
	Deutsch	0.85	0.83	87.77	0.11	93.64	0.08
	Johnson	0.82	0.81	96.08	0	101.95	0
	Lactin	0.85	0.83	88.3	0.09	94.17	0.06
	O'Neill	0.84	0.82	91.29	0.02	97.16	0.01
	Ratkowsky	0.85	0.82	89.06	0.06	94.93	0.04
	Room	0.85	0.83	87.91	0.1	93.78	0.07
	Spain	0.83	0.81	93.42	0.01	99.29	0
	Thébault	0.75	0.77	111.12	0	116.99	0
	van der Heide	0.81	0.8	98.28	0	102.86	0
	Yan and Hunt	0.85	0.82	87.85	0.11	92.43	0.15
Z. muelleri, Summer, Moreton Bay	Briére-1	0.44	0.65	129.53	0	133.74	0
	Briére-2	0.65	0.73	112.85	0.11	118.19	0.11
	Deutsch	0.65	0.73	112.87	0.11	118.21	0.11
	Johnson	0.66	0.74	111.49	0.22	116.82	0.22

Lactin	0.43	0.64	130.93	0	136.27	0
O'Neill	0.66	0.74	113.45	0.08	118.79	0.08
Ratkowsky	0.65	0.73	112.84	0.11	118.18	0.11
Room	0.66	0.74	111.4	0.23	116.74	0.23
Spain	0.35	0.6	138.03	0	143.37	0
Thébault	0.65	0.73	112.36	0.14	117.69	0.14
van der Heide	0.58	0.69	119.22	0	123.42	0.01
Yan and Hunt	0.55	0.68	121.34	0	125.54	0

S4. Supplementary Figures

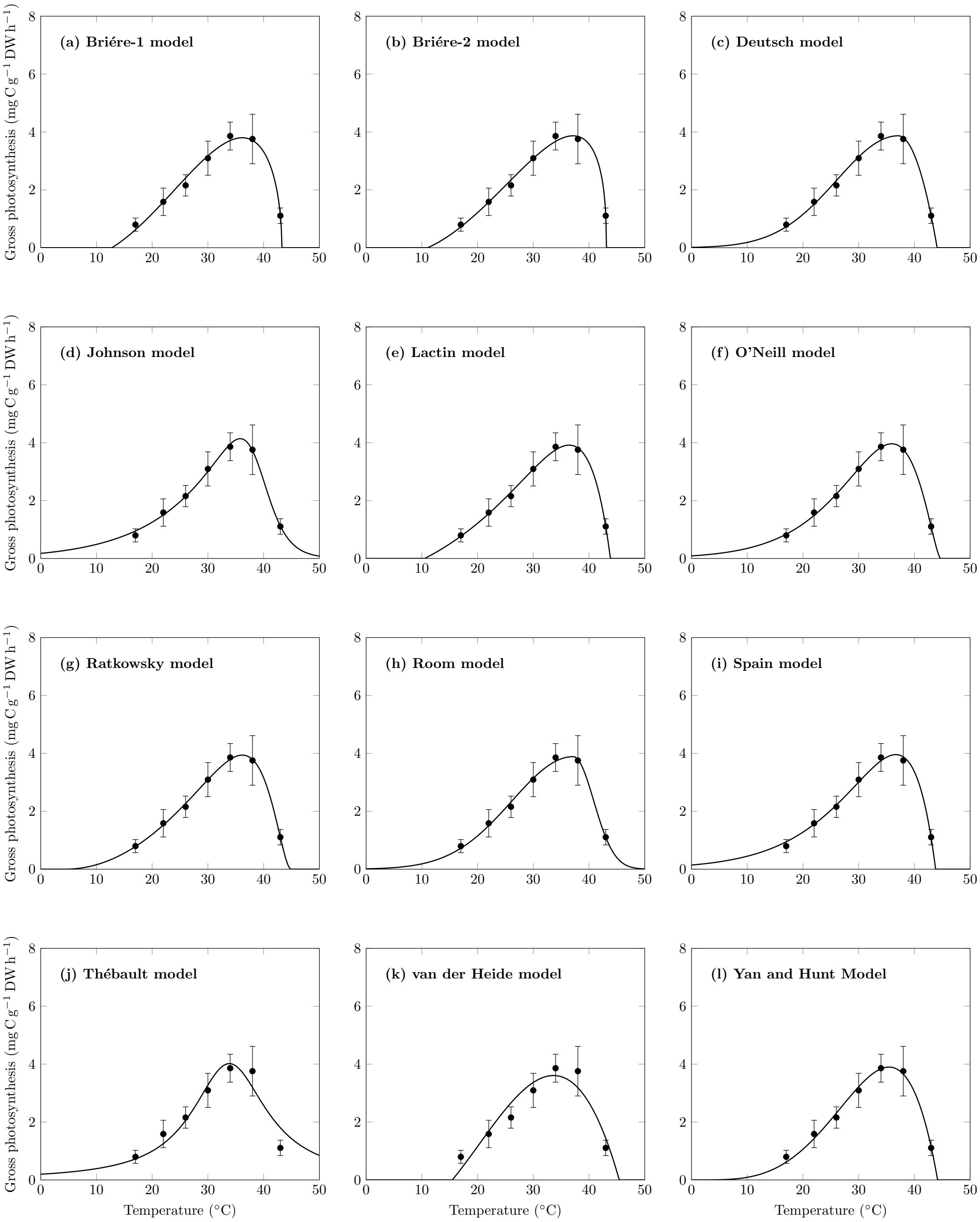

Figure S2: All 12 models fitted to the photosynthesis-temperature curve of *C. serrulata* at Moreton Bay in summer. Error bars indicate ±SD.

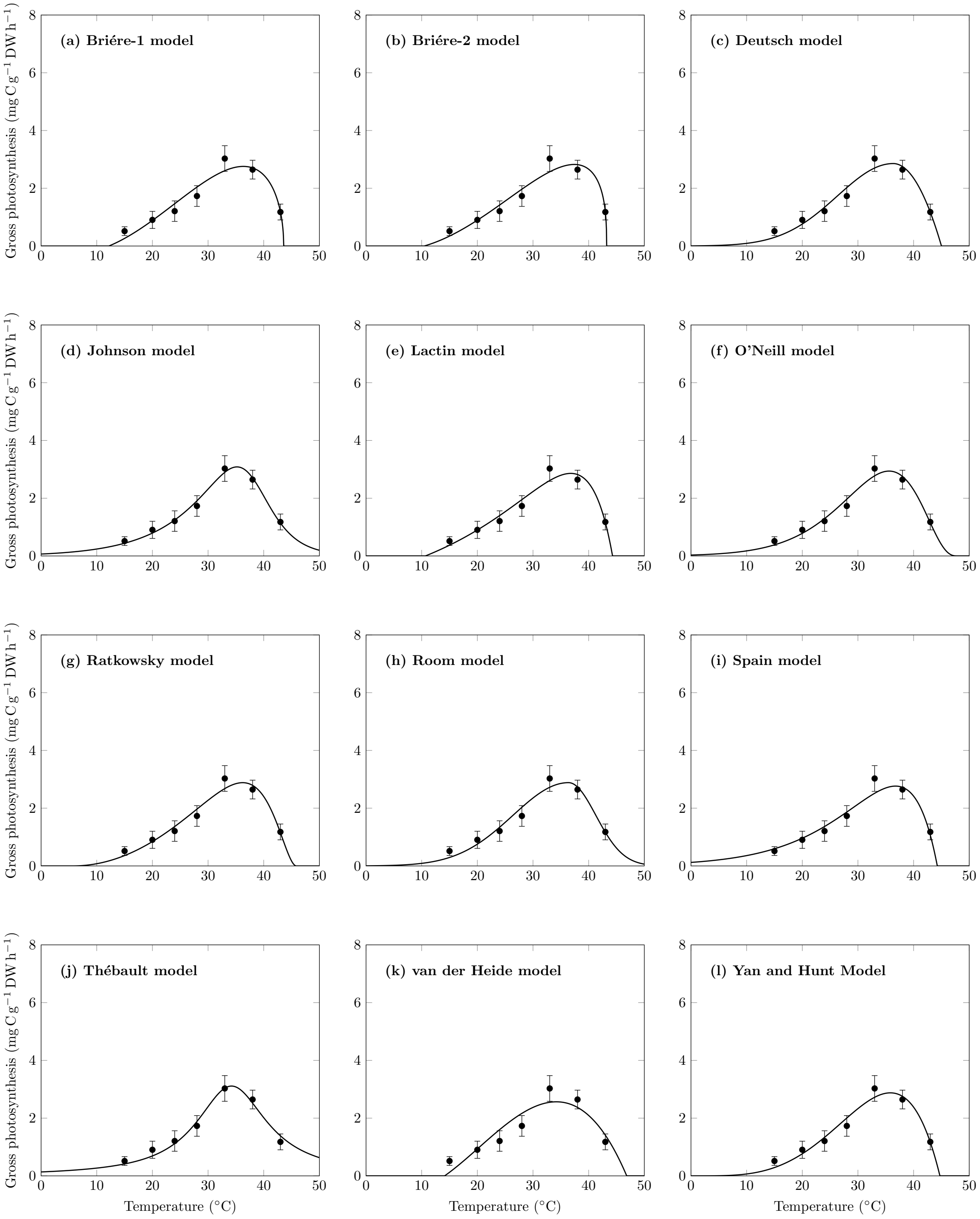

Figure S3: All 12 models fitted to the photosynthesis-temperature curve of *C. serrulata* at Moreton Bay in winter. Error bars indicate ±SD.

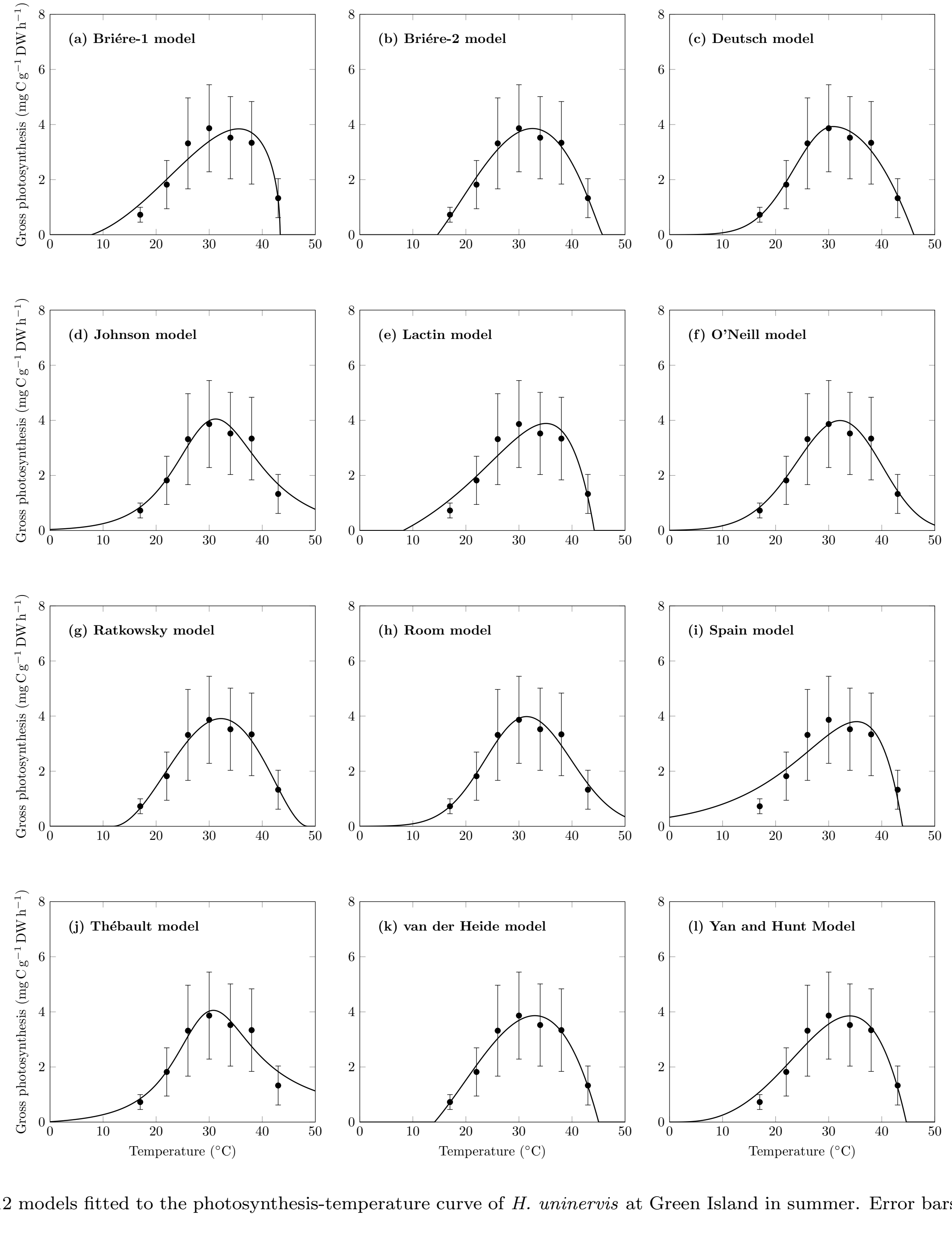

Figure S4: All 12 models fitted to the photosynthesis-temperature curve of *H. uninervis* at Green Island in summer. Error bars indicate ±SD.

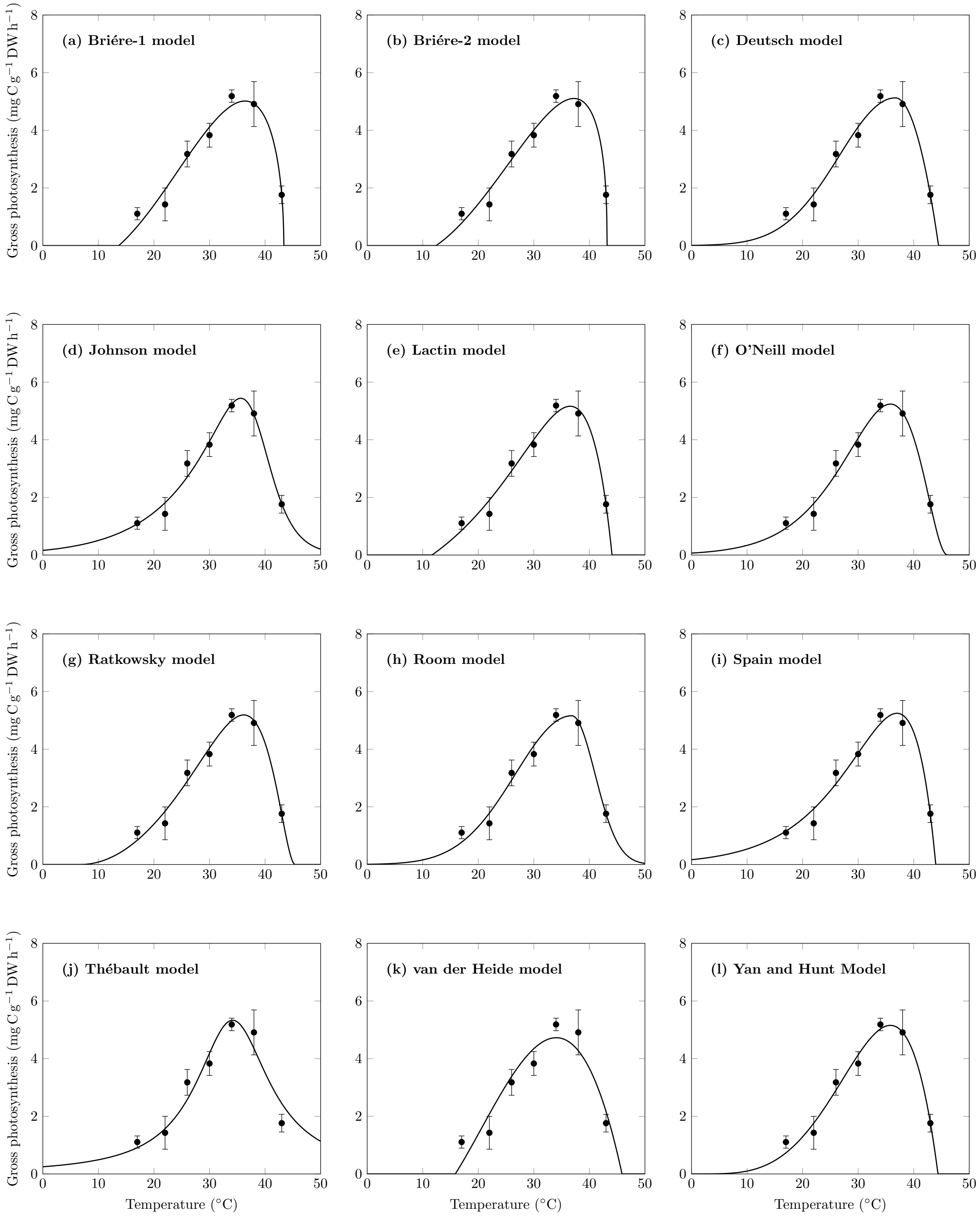

Figure S5: All 12 models fitted to the photosynthesis-temperature curve of *H. uninervis* at Moreton Bay in summer. Error bars indicate ±SD.

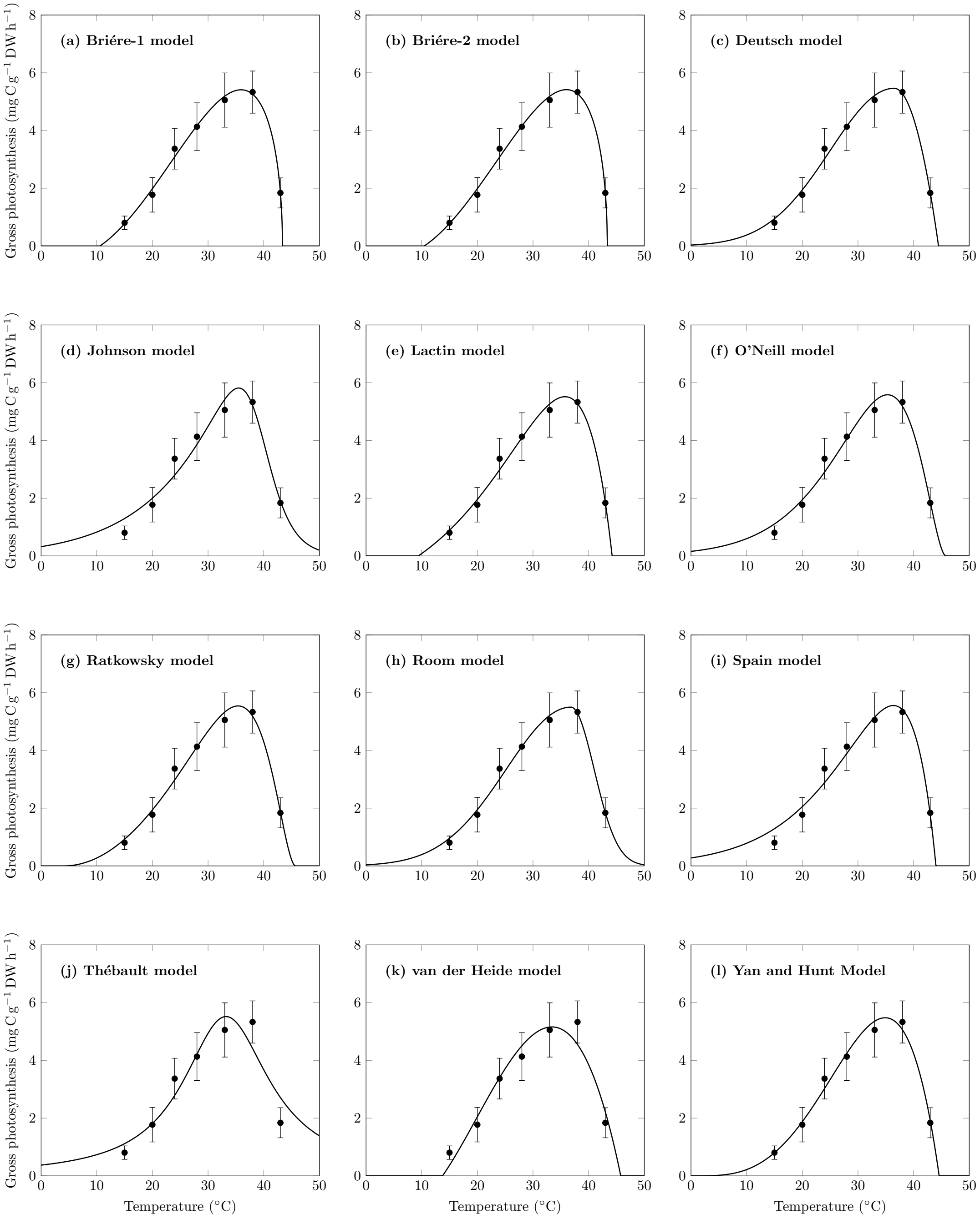

Figure S6: All 12 models fitted to the photosynthesis-temperature curve of *H. uninervis* at Moreton Bay in winter. Error bars indicate ±SD.

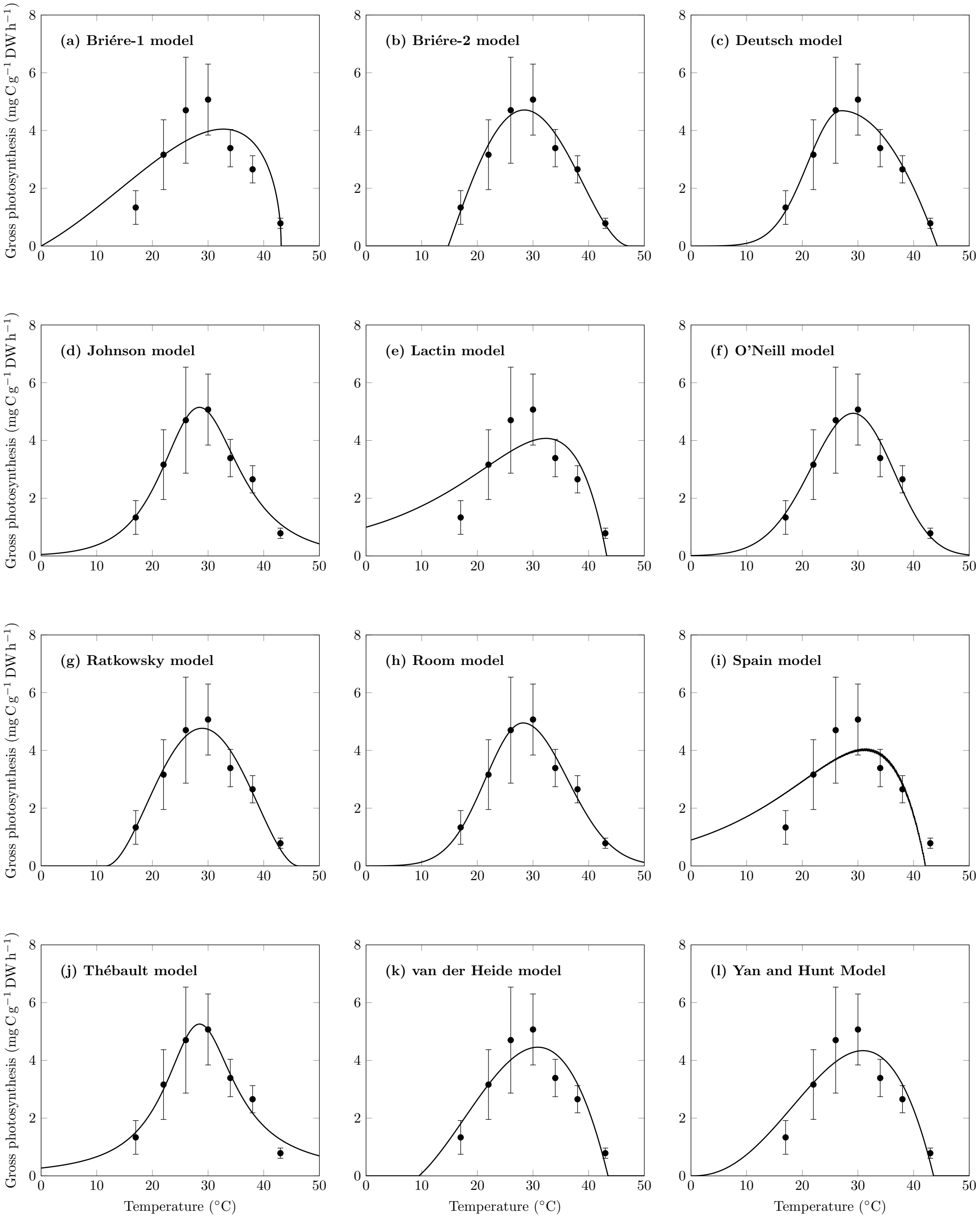

Figure S7: All 12 models fitted to the photosynthesis-temperature curve of *Z. muelleri* at Moreton Bay in summer. Error bars indicate ±SD.

References

Briére, J.-F., Pracros, P., Roux, A.-Y. L., & Pierre, J.-S. (1999). A novel rate model of temperature-dependent development for arthropods. *Environmental Entomology*, *28*, 22–29. doi:10.1093/ee/28.1.22.

Deutsch, C. A., Tewksbury, J. J., Huey, R. B., Sheldon, K. S., Ghalambor, C. K., Haak, D. C., & Martin, P. R. (2008). Impacts of climate warming on terrestrial ectotherms across latitude. *Proceedings of the National Academy of Sciences of the United States of America*, *105*, 6668–6672. doi:10.1073/pnas.0709472105.

van der Heide, T., Roijackers, R. M. M., van Nes, E. H., & Peeters, E. T. H. M. (2006). A simple equation for describing the temperature dependent growth of free-floating macrophytes. *Aquatic Botany*, *84*, 171–175. doi:10.1016/j.aquabot.2005.09.004.

Johnson, F. H., Eyring, H., & Stover, B. J. (1974). *The theory of rate processes in biology and medicine*. New York: John Wiley & Sons, Inc.

Lactin, D. J., Holliday, N. J., Johnson, D. L., & Craigen, R. (1995). Improved rate model of temperature-dependent development by arthropods. *Environmental Entomology*, *24*, 68–75. doi:10.1093/ee/24.1.68.

Li, L., & Yakupitiyage, A. (2003). A model for food nutrient dynamics of semi-intensive pond fish culture. *Aquacultural Engineering*, *27*. doi:10.1016/S0144-8609(02)00037-7.

O'Neill, R. V., Goldstein, R. A., Shugart, H. H., & Manki, J. B. (1972). *Terrestrial ecosystem energy model, US IBP Eastern Deciduous Forest Biome Memo Report 72-19*. Technical Report Oak Ridge National Laboratory, Oak Ridge.

Ratkowsky, D. A., Lowry, R. K., McMeekin, T. A., Stokes, A. N., & Chandler, R. E. (1983). Model for bacterial culture growth rate throughout the entire biokinetic temperature range. *Journal of Bacteriology*, *154*, 1222–1226.

Room, P. M. (1986). Equations relating growth and uptake of nitrogen by *Salvinia molesta* to temperature and the availability of nitrogen. *Aquatic Botany*, *24*, 43–59. doi:10.1016/0304-3770(86)90116-6.

Spain, J. D. (1982). *BASIC microcomputer models in biology*. Reading, MA: Addison-Wesley Publishing Company.

Thébault, J.-M. (1985). Étude expérimentale de la nutrition d'un copépode commun (*Temora stylifera* Dana). Effets de la température et de la concentration de nourriture. *Journal of Experimental Marine Biology and Ecology*, *93*, 223–234. doi:10.1016/0022-0981(85)90241-2.

Yan, W., & Hunt, L. A. (1999). An equation for modelling the temperature response of plants using only the cardinal temperatures. *Annals of Botany*, *84*, 607–614. doi:10.1006/anbo.1999.0955.
